# Oxidation of Allylic Alcohols by Manganese Oxides: Elucidating the Influences of MnO_x_ Structure and Mn Oxidation State on Reactivity

**DOI:** 10.1002/chem.71376

**Published:** 2026-07-20

**Authors:** Erdogan Kasisari, Leandra Meyer, S. Esmael Balaghi, Harald Scherer, Patrick Elsässer, Anna Fischer, Philipp Kurz

**Affiliations:** ^1^ Institut Für Anorganische und Analytische Chemie Albert‐Ludwigs‐Universität Freiburg Freiburg Germany; ^2^ Freiburger Materialforschungszentrum (FMF) Albert‐Ludwigs‐Universität Freiburg Freiburg Germany; ^3^ Freiburg Center for Interactive Materials and Bioinspired Technologies (FIT) Freiburg Germany; ^4^ Cluster of Excellence Living, Adaptive, and Energy‐autonomous Materials Systems (livMatS) Freiburg Germany

**Keywords:** allylic alcohol, heterogeneous oxidation, manganese, oxides

## Abstract

Manganese oxides (MnO_x_) with manganese in oxidation states between +III and +IV are well established in organic synthesis as oxidizing agents for allylic alcohols. In this study, four different Mn^III/IV^O_x_ were synthesized according to established procedures and—together with commercial “activated MnO_2_” – investigated in detail concerning their reactivity in the heterogeneous oxidation of the allylic alcohol *trans*‐pent‐2‐en‐1‐ol. We found that all manganese oxides were able to oxidize *trans*‐pent‐2‐en‐1‐ol to the corresponding aldehyde with excellent selectivities. However, reaction rates differed greatly depending on the oxides’ structures, morphologies, chemical compositions and average Mn oxidation states. Amorphous manganese oxides with layered structures (birnessite‐type) proved to be especially efficient oxidizing agents and the incorporation of Ca^2+^ further increased reactivity. Post‐reaction analyses by X‐ray diffraction and X‐ray spectroscopy indicated that most MnO_x_ materials largely retained their structures and Mn oxidation states, whereas Ca‐birnessite showed clear reaction‐induced structural changes and moderate Mn reduction. Very similar results were obtained for the oxidation of the related substrate cinnamyl alcohol. Overall, it can be concluded that the best oxidation agents for this “classic” of organic synthesis are much more complex materials than simply “MnO_2_” and that the right choice of MnO_x_ is crucial for high product conversions.

## Graphical abstract

Manganese oxides (MnO_X_) of different structures and with different Mn oxidation states exhibit significant variation in their reactivity toward the oxidation of allylic alcohols. Using two allylic alcohol model substrates, we demonstrate the relationship between activity and MnO_X_ structure, Mn^4+^ content and incorporated secondary cations. Layered birnessite‐type manganese oxides, especially calcium birnessite, emerge as highly efficient oxidants.

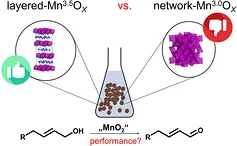

## 1 Introduction

Manganese oxides exhibit a great chemical and structural diversity with more than 30 types of manganese oxides/hydroxides found in the Earth's crust [1]. Prominent examples of these compounds are bixbyite (Mn^III^_2_O_3_), hausmannite (Mn^III^Mn^IV^_2_O_4_), and birnessites. The latter are layered manganese oxides of varying stoichiometries containing both Mn^3+^ and Mn^4+^, often together with s‐block‐metal cations and interlayer water molecules. As shown in Figure 1, these three oxides all feature [Mn^III/IV^O_6_]‐octahedra as central building blocks but strongly differ in the arrangement of these moieties leading to network‐, spinel‐, or layered structures, respectively.

**FIGURE 1:**
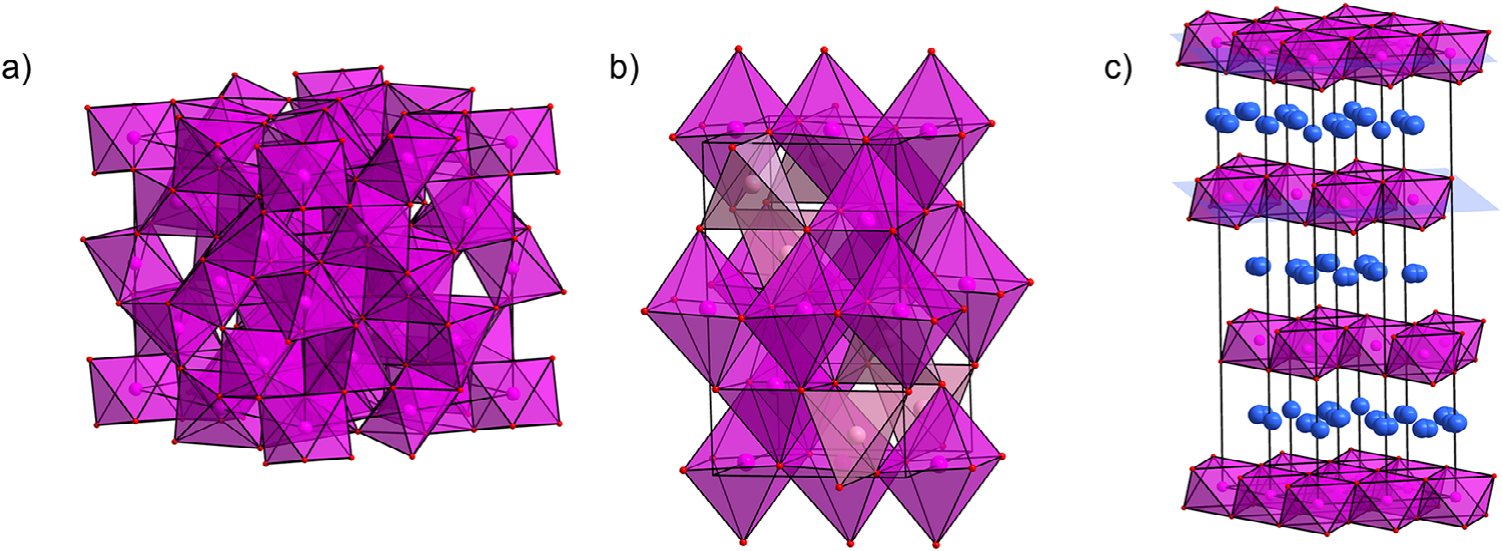
Illustration of different structures found in common manganese oxides: [1] (a) bixbyite (Mn_2_O_3_, network‐structure), [2] (b) hausmannite (Mn_3_O_4_, spinel‐structure), [3] and (c) birnessite (here: layered structure of synthetic potassium birnessite) [4]. Color code: red = oxygen (O of O^2−^ or OH^−^); [Mn^III/IV^O_6/4_]‐ octahedra/tetrahedra in magenta/light magenta; blue = K^+^ or water.

As investigated extensively by some of us and others, manganese oxides are well suited to act as (electro)catalysts for the water oxidation reaction, a process that is of enormous technical but also biological importance where it is catalyzed by the CaMn_4_O_5_‐active site of photosystem II [5]. Furthermore, manganese oxides have a variety of applications in organic synthesis (Scheme 1). In addition to the probably best‐known chemoselective oxidation of primary and secondary allylic alcohols to aldehydes (**1**, **2**), there are numerous other reactions involving MnO_x_ which lead to the syntheses of propargylic alcohols (**3**), [6] carboxylic acid amides (**4**), [7] imines (**5**), [8] heteroaromatic rings (**6**), [9], and even esters (**7**) [10]. Decarboxylation reactions via iminium intermediates (**8**) [11] are another possible application. Due to this variety of reactions, there are also many commercial applications of MnO_x_‐based oxidations in the chemical industry. A prominent example is the oxidation of the allylic alcohol retinol (provitamin A) by manganese oxides to retinal (vitamin A) [12], a compound with numerous applications in both the cosmetics and pharmaceutical industries [13].

**SCHEME 1:**
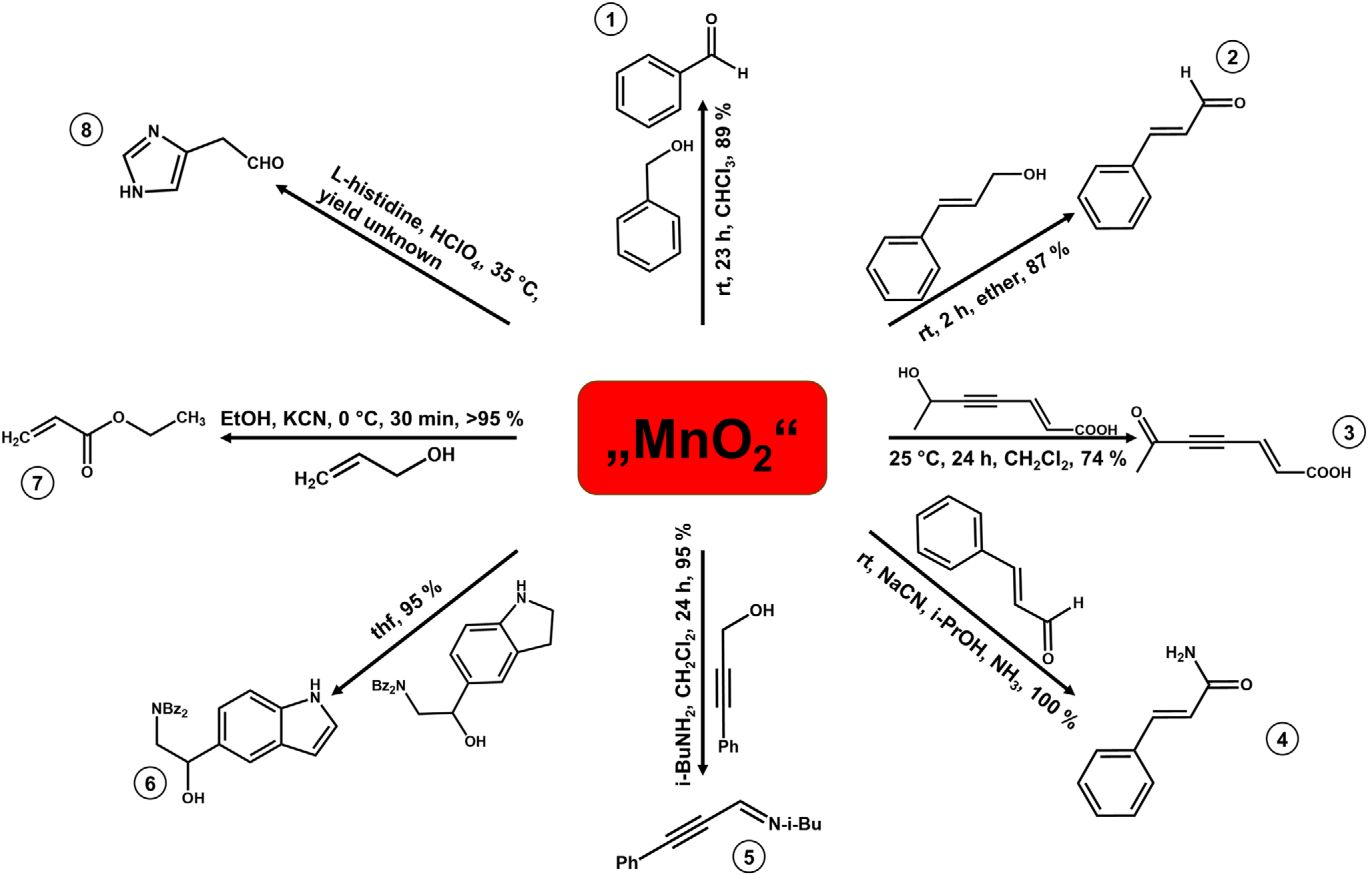
Examples of the diverse reactions where manganese oxides are used in organic synthesis: oxidations of benzyl alcohols (**1**), allylic alcohols (**2**) [16], propargylic alcohols (**3**) [6], syntheses of carboxylic acid amides (**4**) [7], imines (**5**) [8], aromatic heterocycles (**6**) [9], esters (**7**) [10], as well as a decarboxylation reaction (**8**) [11].

Despite the well‐known large structural diversity of manganese oxides mentioned above, it is striking that in most organic synthesis protocols and textbooks the manganese oxide reagent is simply given as “MnO_2_”, although it is clear from the experimental details that almost never pure, crystalline manganese(IV) oxide is employed [6, 7, 8, 9, 10, 11, 14]. Some authors admit that the used manganese oxides are “nonstoichiometric hydrated material[s]” [14], but no further details about these are given.

Consequently, we decided that a closer look at the manganese oxides’ structures, compositions, and reactivities could be beneficial to this field of well‐established organic transformations and decided to investigate the oxidation of the allylic alcohol *trans*‐pent‐2‐en‐1‐ol by different manganese oxides as a model reaction (Scheme 2). For our study, we chose oxides that had been reported to be highly reactive either for the oxidation of organic molecules or in water oxidation catalysis. In preparative organic chemistry, a procedure published by Attenburrow et al. already in 1952 is often used to obtain chemically active manganese oxides [15]. For this reason, we synthesized this manganese oxide as a reference compound for oxidation activity. Additionally, to our knowledge a detailed characterization of this material has so far not been carried out and could therefore be advantageous for a better understanding of this established MnO_x_ reagent. Furthermore, synthetic K^+^‐ and Ca^2+^‐ containing birnessites were chosen because of their well‐known good performances in water oxidation (electro)catalysis [5]. Finally, a commercial “manganese dioxide” sold explicitly for use as oxidation agent in organic synthesis, and synthetic bixbyite as a compound with a clearly defined Mn oxidation state and structure were added to our list of investigated manganese oxide materials.

**SCHEME 2:**
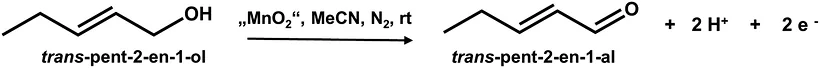
Oxidation of *trans*‐pent‐2‐en‐1‐ol using manganese oxides.

For the study, four manganese oxides were synthesized following established procedures and then (together with the commercial MnO_2_) carefully characterized before and after their reactions with *trans*‐pent‐2‐en‐1‐ol by a variety of analytical methods. Ion chromatography (IC) was used in combination with redox titrations to determine the chemical compositions and the average manganese oxidation states for the samples. To gain further insights into the atomic structures, oxidation states, crystallinities and surface areas of the manganese oxides, powder X‐ray diffraction (pXRD), infrared spectroscopy (IR), Xp‐ray absorption spectroscopy (XANES and EXAFS) and surface area measurements by nitrogen physisorption were carried out. The reaction conversions, kinetics, and selectivities of the alcohol oxidations were investigated using nuclear magnetic resonance spectroscopy (NMR). By combining this large variety of analytical methods, we aimed to present a thorough analysis of the influence of both the MnO_x_ structure and the Mn oxidation state on the reactivity of such compounds as oxidants in organic synthesis exemplified by the oxidation of an allylic alcohol.

## 2 Results and Discussion

### 2.1 Alcohol Oxidation Reactions

After a basic chemical and structural characterization of the five selected MnO_x_ (see below), we carried out two series of experiments to obtain information about the oxidation reaction of *trans*‐pent‐2‐en‐1‐ol (Scheme 2) in general and possible differences of the reactivities and product selectivities of the five oxides in particular.

First, we had noted that a potential major disadvantage of these reactions is the high excess in which the MnO_x_ species are usually added. We thus first tried to identify the amount of manganese oxide really required for the full conversion of *trans*‐pent‐2‐en‐1‐ol in acetonitrile for the case of the established Attenburrow oxide. As expected, the conversion of the reaction strongly depends on the amount of manganese oxide used (Figure 2). Following the conversion of alcohol to aldehyde by NMR for a total of 100 min, we found that the addition of the Attenburrow‐MnO_x_ in a molar Mn:alcohol‐ratio of 10:1 is necessary to achieve a quantitative conversion. Reactions of only 5 or 1 molar equivalents of Mn per *trans*‐pent‐2‐en‐1‐ol led to only 70% or 10 % substrate conversions, respectively. All reactions follow pseudo‐first order kinetics depending linearly on the *trans*‐pent‐2‐en‐1‐ol concentration and require about 1 h at room temperature to reach their respective maximum conversions. The NMR analysis of the product mixtures revealed in all cases the well‐known superb selectivity of the oxidation process, as *trans*‐pent‐2‐en‐1‐al was the only detected reaction product for all three Mn:alcohol‐ratios at any of the investigated reaction times (for sample spectra and details on the NMR quantification protocol, see ESI, Figures S1–S5).

**FIGURE 2:**
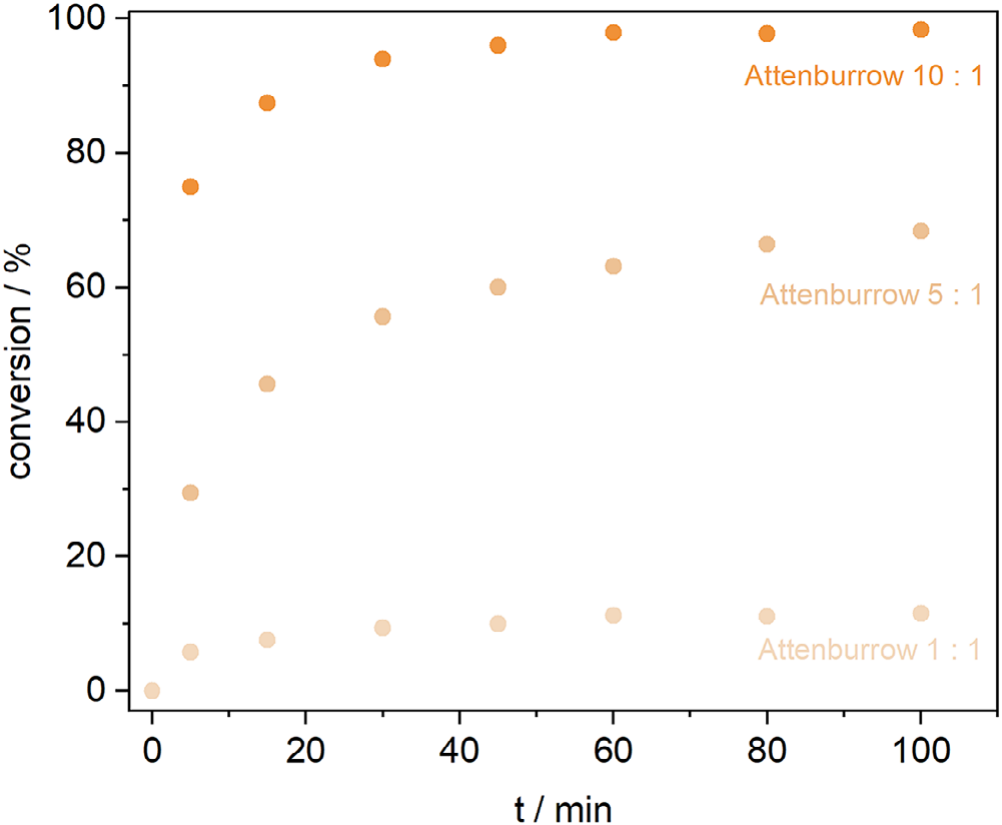
Time‐dependent conversion of *trans*‐pent‐2‐en‐1‐ol to *trans*‐pent‐2‐en‐1‐al in N_2_‐saturated acetonitrile at room temperature using various Mn:substrate ratios of Attenburrow‐MnO_x_ (10:1, 5:1, and 1:1).

Having established that a molar Mn:alcohol ratio of 10:1 is indeed beneficial for the oxidation of *trans*‐pent‐2‐en‐1‐ol to *trans*‐pent‐2‐en‐1‐al, we now followed the reactions of this alcohol with the other four selected MnO_x_, always applying a tenfold excess of Mn per substrate molecule. Here, we observed dramatic differences in reactivity between the various manganese oxides (Figure 3). Although some conversion of the alcohol to the aldehyde can be achieved with all five oxides, the oxide's reactivities vary strongly and the following general trend was observed:

**FIGURE 3:**
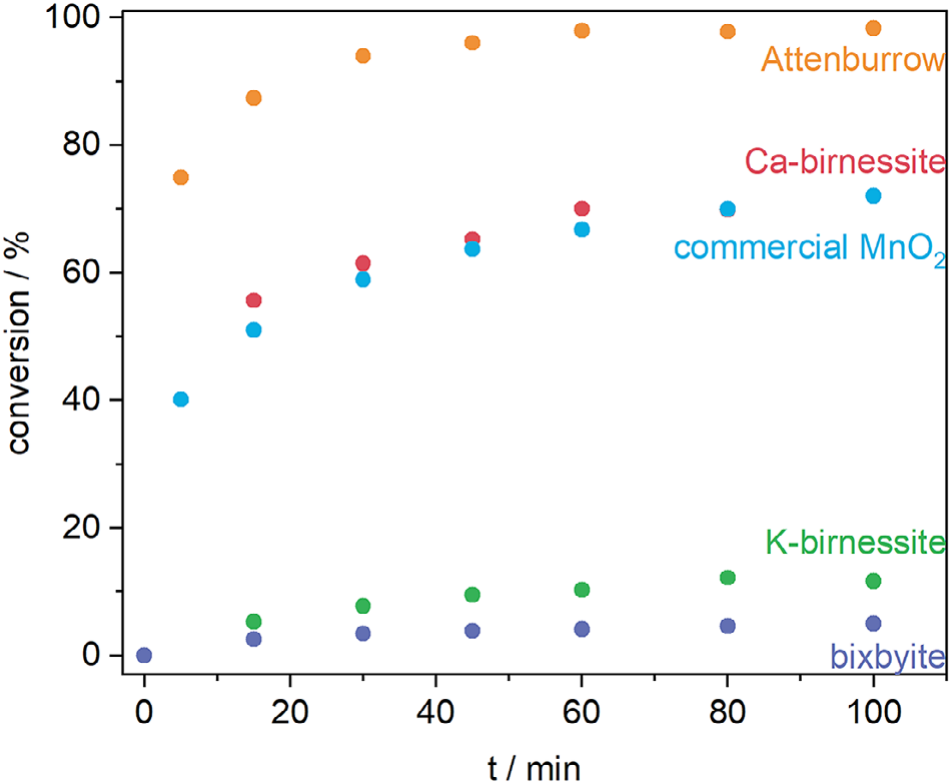
Time‐dependent conversion of *trans*‐pent‐2‐en‐1‐ol to *trans*‐pent‐2‐en‐1‐al in N_2_‐saturated acetonitrile with different manganese oxides. The Mn:substrate ratio was 10:1 in all cases.

Attenburrow‐MnO_x_ > Ca‐birnessite ≈ commercial MnO_2_ >> K‐birnessite > bixbyite

The “right” choice of manganese oxide therefore seems decisive for a successful application as oxidation agent for the allylic alcohol *trans*‐pent‐2‐en‐1‐ol. In particular, the presence of Mn^IV^ seems to be beneficial, but also the greatly accelerated reaction when exchanging potassium with calcium in birnessites is remarkable and will be discussed in detail below. On the other hand, the chemoselectivity of the reaction is excellent for all five tested MnO_x_, as *trans*‐pent‐2‐en‐1‐al is again the only NMR‐detected product (see ESI, Figures S6–S9). This leads to the conclusion that the redox potential of MnO_x_ is for all five MnO_x_ sufficient for the oxidation of the alcohol to the aldehyde, while the further reaction to the carboxylic acid is not observed.

As the reaction also generates protons as a reaction product (Scheme 2), the influence of water and base on the reaction rates and yields was also investigated. For the Attenburrow‐MnO_x_, we found that already an addition of 10 vol.% of water to the acetonitrile solvent leads to a large decrease of the product yield from ∼95% after 60 min. in pure MeCN to only ∼13% in MeCN:H_2_O  =  9:1. Similarly, the addition of 10 vol.% of a 0.1 M aqueous sodium carbonate solution to the acetonitrile was also not beneficial (see ESI, Figure S10). This may be explained by the interaction of water with the manganese oxide, where surface sites otherwise available for substrate‐binding are now blocked by water molecules thus acting as a poison for the catalyst. Other working groups have already reported a strong influence of the solvent on the reactivity of manganese oxides in similar reactions [17, 18].

### 2.2 Manganese Oxide Structures

Powder X‐ray diffraction (pXRD) with copper radiation (*λ* =  154.187 pm) was used as first analytical tool to gain insights into the structures of the different manganese oxides both before and after their reactions with *trans*‐pent‐2‐en‐1‐ol (Figure 4). Here, the very weak and broad reflections detected for the Attenburrow oxide, the commercial “MnO_2_” and the synthetic Ca‐birnessite indicate that all three are rather amorphous materials lacking long‐range order.

**FIGURE 4:**
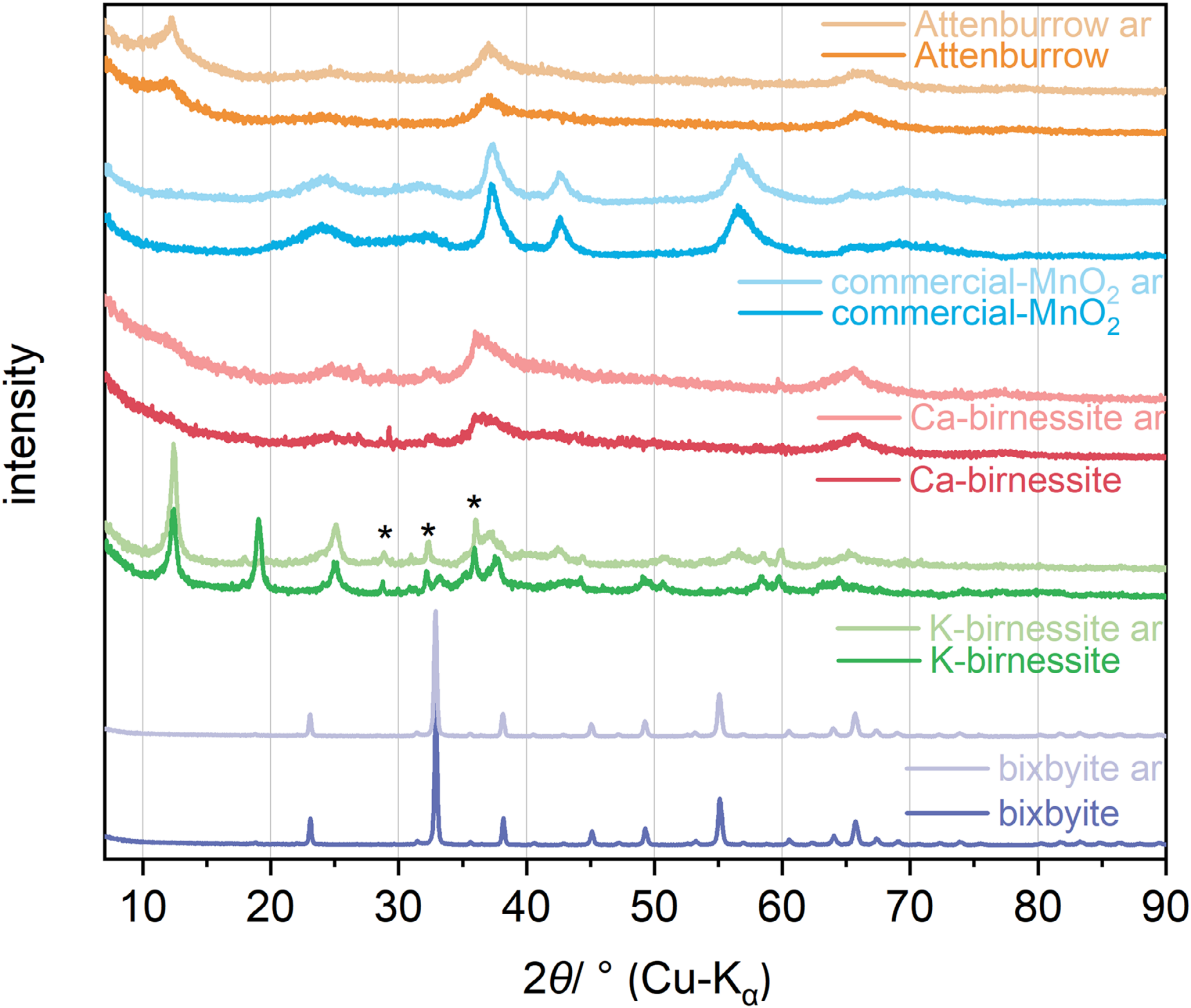
pXRD patterns of the investigated manganese oxides before and after their reactions with *trans*‐pent‐2‐en‐1‐ol (Mn : substrate ratio 10 : 1, reaction time 120 min.). In all cases, the less intense color indicates the MnO_x_ species after its reaction with *trans*‐pent‐2‐en‐1‐ol. Reflexes most likely originating from hausmannite (Mn_3_O_4_) impurities are marked with an asterisk (*).

On the other hand, the diffractograms of bixbyite and K‐birnessite both feature intense reflections and allow a clear identification of these oxides as network‐ or layered structures, respectively (Figure 1). The layered structure of the K‐birnessite is well indicated by a reflection at *2θ*  = 12.5 , which can be traced back to the (001) plane, and one at *2θ*  = 19° originating from the second‐order (002) diffraction. This reflex position can also be used to determine the distance between the [MnO_6_]‐layers in the structure accounting for ∼7.3 Å, which is a typical value for K‐birnessites [19]. Additional signals at ∼19° and ∼25° in the diffractogram of the K‐birnessite are also well‐known for birnessite‐type manganese oxides [20]. The pXRD data additionally indicates that the K‐birnessite prepared for this study is not phase‐pure but shows some additional reflexes that can be assigned to hausmannite (Mn_3_O_4_).

After their reactions with *trans*‐pent‐2‐en‐1‐ol for 2 h, the oxides were recovered by filtration, dried, and analyzed again using pXRD (see ESI for details). In all five cases, the diffractograms remain virtually identical when comparing the data before and after the reactions (Figure 4).

Next, we took a close look at the ATR–IR spectra obtained for all five oxides before and after the reaction with the allylic alcohol (Figure 5). In all of these, we find the typical symmetrical vibrations of [MnO_6_]‐octahedra in the region of 750–500 cm^−1^, [21] confirming the presence of this common building block for all five studied manganese oxides both before and after their reactions with the allylic alcohol. The vibrational spectra of the bixbyite and the commercial MnO_2_ only show these [MnO_6_]‐signals and little change of them after reaction. In contrast, additional broad IR bands can be detected for the Attenburrow–MnO_x_ and the K‐birnessites between 3600 cm^−1^ and 3000 cm^−1^, which can be assigned to OH groups or water molecules bound within the structures or on the oxides’ surfaces [22]. It is interesting to note that for these two oxides this feature decreases in intensity after the reaction with the strongest effect visible for the Attenburrow–MnO_x_.

**FIGURE 5:**
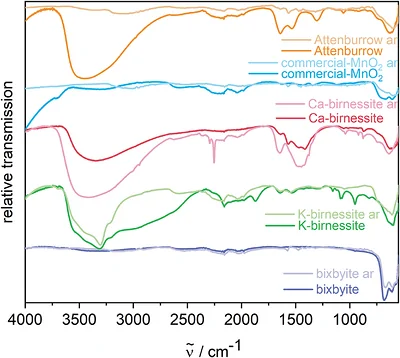
ATR–IR spectra of Mn‐oxides before and after their reactions with *trans*‐pent‐2‐en‐1‐ol (Mn:substrate ratio 10:1, reaction time 120 min.). Violet: bixbyite, green: K‐birnessite, red: Ca‐birnessite, blue: commercial MnO_2_, orange: Attenburrow–MnO_x_. In all cases, the less intense color indicates the MnO_x_ species after its reaction with *trans*‐pent‐2‐en‐1‐ol.

In the case of the IR‐spectrum for Ca‐birnessite after the reaction, prominent new signals appear at ∼2250 cm^−1^ and between 1500 cm^−1^ and 1300 cm^−1^. As this sample had to be dried at much milder conditions than the others (see ESI for details), it is possible that these bands originate from acetonitrile solvent or the reaction product *trans*‐pent‐2‐en‐1‐al still bound to the oxide's surface (see ESI, Figure S11). Actually, “free” carbonyls show signals at 1850–1600 cm^−1^ [22], but the observed shift to lower vibration energy might be explained by a back‐bonding interaction of the Mn d electrons with the π*‐orbitals of the aldehyde's carbonyl moiety [23].

Extended X‐ray absorption fine structure (EXAFS) data can provide further insights into the local Mn coordination geometries and changes in the Mn─O─Mn networks [24]. X‐ray absorption spectroscopy (XAS, see below) measurements were carried out using an in‐house EasyXAFS300^+^ X‐ray absorption spectrometer with samples kept at ambient conditions to probe structural and oxidation‐state changes resulting from the reaction of the MnO_x_ with *trans*‐pent‐2‐en‐1‐ol. The Fourier‐transformed EXAFS spectra of all MnO_x_ show a dominant first‐shell peak at an apparent (phase‐uncorrected) radial distance *R*′ ≈ 1.5 Å, corresponding to Mn─O bonds (Figure 6). This equals a real‐space Mn─O distance of ∼1.9 Å, typical for MnIII/IV─O in octahedral, all‐oxygen coordination environments [25, 26]. The offset between R′ and d(Mn─O) results from the photoelectron phase shift at the Mn K‐edge [27, 28].

**FIGURE 6:**
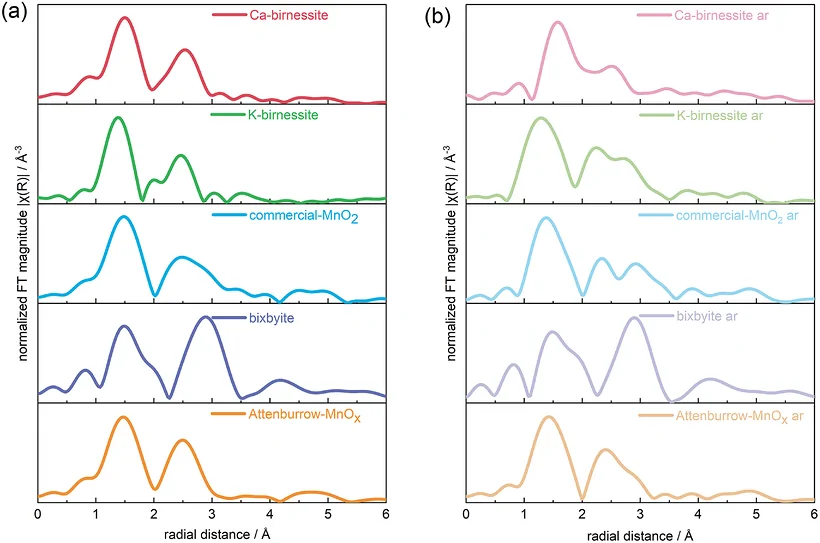
Mn K‐edge FT‐EXAFS of MnO_x_ (a) before and (b) after their reactions with *trans*‐pent‐2‐en‐1‐ol (“ar”, Mn:substrate ratio 10:1, reaction time 120 min.). The Fourier transforms of k^2^χ(k) (*k* = 3–11 Å^−^^1^; Hanning window, dk = 1) are plotted as |χ(R)| versus the phase‐uncorrected radial distances R’. Traces are identically normalized and plotted with identical y‐axis limits. The first‐shell Mn─O contributions appear at an apparent *R*’ of ∼1.5–2.0 Å, while the edge‐sharing Mn─Mn features are found at R’ ∼ 2.8–3.0 Å. Background subtraction and normalization were performed as described in the methods section (see ESI for details).

In the second‐shell region (*R*′ ∼2.5–3.5 Å), clear differences emerge between the structures of the different oxides. The Attenburrow oxide, the K‐/Ca‐birnessites and the commercial MnO_2_ all exhibit a prominent peak around *R*′ ∼2.8–3.0 Å, attributable to Mn─Mn scattering from edge‐sharing [MnO_6_] octahedra within MnO_x_ layers [24]. This is a hallmark of layered birnessite‐type structures. In contrast, bixbyite (Mn_2_O_3_) shows a different FT‐EXAFS fingerprint. Its second‐shell features are shifted to a higher distance with a notable peak beyond 3 Å, corresponding to the longer Mn─Mn separations due to the corner‐sharing arrangement of the [MnO_6_]‐octahedra in the bixbyite lattice (Figure 1) [29].

To benchmark distances and disorder parameters, we applied a quantitative EXAFS fit to the Ca‐birnessite data using FEFF, an ab initio multiple‐scattering code used to calculate theoretical EXAFS scattering paths. The fit used a birnessite model structure with split Mn‐O subshells and in‐plane edge‐sharing Mn─Mn paths (see ESI, Figure S12). The spectrum is reproduced well (S_0_^2^ fixed at 0.92), yielding Mn─O_short_ ≈ 1.94 Å, Mn─O_long_ ≈ 2.03 Å with a small long‐bond fraction, and Mn─Mn ≈ 2.79/2.87 Å, in agreement with earlier analyses of Ca‐containing birnessites [24, 30]. For the other MnO_x_, we restrict the discussion to the qualitative FT features: both Attenburrow‐MnO_x_ and commercial “MnO_2_” exhibit broadened/weaker second‐shell peaks (lower order, distributed Mn─Mn distances), whereas K‐birnessite and bixbyite retain sharper second‐shell structure.

After their reactions with *trans*‐pent‐2‐en‐1‐ol, the EXAFS spectra reveal the extent of structural changes. For Attenburrow‐MnO_x_, only minor modifications are visible: the already broad second‐shell features decrease slightly in amplitude and the ∼2.9 Å peak weakens, consistent with the loss of some Mn─Mn connections (surface amorphization and/or removal of interlayer water/hydroxide). The commercial MnO_x_ and the K‐birnessite behave similarly, retaining their overall structures with only slight attenuation of higher‐shell features, correlating with the minimal XRD changes (Figure 4).

In marked contrast, Ca‐birnessite shows a pronounced reaction‐induced transformation. Beyond the first shell, the FT magnitude is clearly diminished and the birnessite‐type edge‐sharing Mn─Mn feature at *R*’ ∼2.8–3.0 Å is strongly attenuated and broadened, indicating a partial loss of the birnessite‐type in‐plane order. Additional intensity appears at slightly higher *R*’ of ∼3.1–3.4 Å, which is compatible with a partial reorganization into a spinel/hausmannite‐like structure. These EXAFS changes, together with the XANES evidence for moderate Mn reduction (see below), indicate that the highly active, defect‐rich, and Ca^2+^‐intercalated Ca‐birnessite undergoes partial reduction and local structural reorganization during its reaction with the allylic alcohol. The material post‐reaction is therefore best described as a mixed and disordered structure containing residual birnessite‐type motifs and partial spinel/hausmannite‐like domains, rather than as a fully collapsed or completely transformed phase.

Finally, the bixbyite sample exhibits a modest reduction in the amplitudes of the ∼2.9 Å and ∼3.4 Å Mn─Mn peaks after reaction, pointing to slightly increased disorder and a small loss of Mn neighbors (e.g., oxygen vacancies or minor Mn^II^─O domains as a result of partial Mn^III^ reduction). The bixbyite structure is largely retained, as also evidenced by the essentially unchanged pXRD pattern shown above (Figure 4).

To wrap up the EXAFS analysis, we compared the post‐reaction FT‐EXAFS spectra with those of reference materials (see ESI, Figure S13). Here it is evident that the spectral changes described above for the Ca‐birnessite sample after reaction are not consistent with a simple MnO_2_‐like structure, where the edge‐sharing Mn─Mn feature is largely retained, and it is also not reproduced by Mn_2_O_3_ alone, which is dominated by higher‐R corner‐sharing Mn‐Mn contributions at *R*’ > 3.2 Å. Because of the described mixed character, multi‐shell EXAFS fits would not be unique for such a material and we therefore did not carry out a quantitative fitting in the case of Ca‐birnessite ar. For the other oxides, the reference spectra confirm the trends observed in Figure 6: K‐birnessite and commercial MnO_2_ largely preserve their edge‐sharing Mn‐Mn features with only minor damping, bixbyite retains its corner‐sharing contributions at *R*’ > 3.2 Å and the Attenburrow‐MnO_x_ mainly shows broadening of its already disordered higher‐shell features. A more detailed comparison of the Ca‐birnessite data both before and after reaction and including FT‐EXAFS, XANES, and WT‐EXAFS maps is provided in Section 7 of the ESI.

### 2.3 Changes of the Cation Compositions and the Average Manganese Oxidation States

After dissolving samples of the oxides in a HNO_3_/H_2_O_2_ mixture, it was possible to determine the Na^+^‐, K^+^‐, Ca^2+^‐, and Mn^n+^‐ contents for the oxides using ion chromatography (IC). Additionally, redox back‐titrations were carried out after the different oxides had reacted with oxalic acid (see Experimental Section for details on both methods). In combination, the data obtained from the IC measurements and the titrations were then used to calculate the chemical compositions of the oxides (Table 1) as well as the average oxidation states of manganese for all five MnO_x_ both before and after their reactions with *trans*‐pent‐2‐en‐1‐ol (Table 2).

**TABLE 1:** Cation composition of the manganese oxides (molar ratios per Mn). Chemical formulae in the last column were obtained by combining ion chromatography (Na^+^/K^+^/Ca^2+^/total Mn after HNO_3_/H_2_O_2_ digestion) with redox back‐titration for O stoichiometry; waters of hydration are reported as “·xH_2_O”. Reported values are rounded to two decimals (typical IC uncertainty ±5%–10% rel.). Sample names match the rest of the manuscript. Values below the IC detection limit are listed as 0.00.

Compound	Mn	K	Ca	Na	Chemical formula
Attenburrow‐MnO_x_	1.00	0.13	0.00	0.27	K_0.1_Na_0.3_MnO_2.2_·0.5 H_2_O
Attenburrow‐MnO_x_ ar	1.00	0.12	0.00	0.27	K_0.1_Na_0.3_MnO_2.1_
Ca‐birnessite	1.00	0.19	0.21	0.00	K_0.2_Ca_0.2_MnO_2.3_·0.6 H_2_O
Ca‐birnessite ar	1.00	0.16	0.20	0.00	K_0.2_Ca_0.2_MnO_2.1_·0.1 H_2_O
Commercial MnO_2_	1.00	0.00	0.05	0.00	Ca_0.1_MnO_2.0_
Commercial MnO_2_ ar	1.00	0.00	0.00	0.00	MnO_1.9_
K‐birnessite	1.00	0.17	0.00	0.00	K_0.2_MnO_1.7_·0.3 H_2_O
K‐birnessite ar	1.00	0.19	0.00	0.00	K_0.2_MnO_1.8_·0.2 H_2_O
Bixbyite	1.00	0.00	0.00	0.00	MnO_1.5_
Bixbyite ar	1.00	0.00	0.00	0.00	MnO_1.6_

**TABLE 2:** Average manganese oxidation states of the MnO_x_ samples as determined by redox titrations and XAS; S_BET_ values from N_2_‐physisorption; conversions of *trans*‐pent‐2‐en‐1‐ol to *trans*‐pent‐2‐en‐1‐al after 60 min from quantitative ^1^H NMR. ar denotes the material after reaction with *trans*‐pent‐2‐en‐1‐ol (Mn:substrate = 10:1, 120 min); n.d. = not determined due to measurement‐time constraints.

	Average Mn oxidation state			
Compound	From redox titrations	From XANES spectrum	*S*_BET_ (m^2^/g)	*trans*‐Pent‐2‐en‐1‐ol conversion [%]
Attenburrow‐MnO_x_	4.0	4.0	97	
Attenburrow‐MnO_x_ ar	3.8	4.1	n.d.	∼100
Attenburrow‐MnO_x_ (5:1) ar	3.9	n.d.	n.d.	65
Attenburrow‐MnO_x_ (1:1) ar	3.8	n.d.	n.d.	10
Ca‐birnessite	3.9	4.0	255	
Ca‐birnessite ar	3.7	3.7	n.d.	70
Commercial MnO_2_	3.9	4.0	91	
Commercial MnO_2_ ar	3.9	4.2	n.d.	70
K‐birnessite	3.3	3.1	50	
K‐birnessite ar	3.5	3.3	n.d.	10
Bixbyite	3.0	3.0	11	
Bixbyite ar	3.2	2.8	n.d.	5

The results listed in Table 1 support the previously mentioned trends. First, it was confirmed that layered, amorphous oxides like the Attenburrow‐MnO_x_ and the two birnessites contain substantial amounts of Na^+^, K^+^, and/or Ca^2+^ as secondary cations. These cations remain part of the solids also after the reaction with *trans*‐pent‐2‐en‐1‐ol, in line with the generally observed structural stability of these materials. Secondly, the compositions indicate a loss of water for Attenburrow‐MnO_x_ and for both birnessites during the reaction—consistent with the ATR‐IR results and the fact that acetonitrile was used as solvent.

In its second column, Table 2 lists the average manganese oxidation states found by the redox titration method for all studied oxides before and after reaction. As the alcohol oxidation is a two‐electron process (Scheme 2) and the Mn:alcohol ratio was 10:1, one would expect the average Mn oxidation state to decrease by a maximum of 0.2 units if complete alcohol oxidation occurred. For Attenburrow‐MnO_x_ and Ca‐birnessite, we indeed observe decreases of about 0.2 units. When the stoichiometry is lowered to a Mn:substrate ratio of 5:1, the alcohol conversion drops to ∼63% (see Figure 2 above) and the corresponding decrease in the average Mn oxidation state is now only ∼0.1, as expected. For the other oxides, however, the picture is less consistent. For the commercial MnO_2_, the average oxidation state remains unchanged although two thirds of the allylic alcohol are converted; for K‐birnessite and bixbyite the apparent average oxidation state even increases despite low conversions.

We therefore turned to the X‐ray absorption data again, this time analyzing the X‐ray absorption near‐edge structure (XANES) region of the spectra (Figure 7). XANES has been routinely used to determine Mn average oxidation states (AOS) in sample types ranging from solid‐state materials to manganese metalloproteins. The Mn K‐edge XANES spectra of all MnOx (besides bixbyite) before reaction are indicative of mixed Mn^III/IV^ oxidation states and octahedral coordination environments: the edge rises are smooth and largely featureless with low‐intensity pre‐edge peaks, as typical for birnessite‐type oxides that predominantly contain Mn^IV^ in edge‐sharing [MnO_6_]‐octahedra [24]. The edge positions (half‐height energies) reflect average Mn valences >3.0 for all samples except bixbyite (∼3.0). A comparison of the Mn AOS derived from XANES with those from the redox titrations (Table 2) shows an overall good agreement concerning the absolute values. Small differences of ∼0.1–0.2 units are expected and are ascribed to the combined accuracy limits of the two methods when applied to these complex, partly non‐stoichiometric MnO_x_ materials.

**FIGURE 7:**
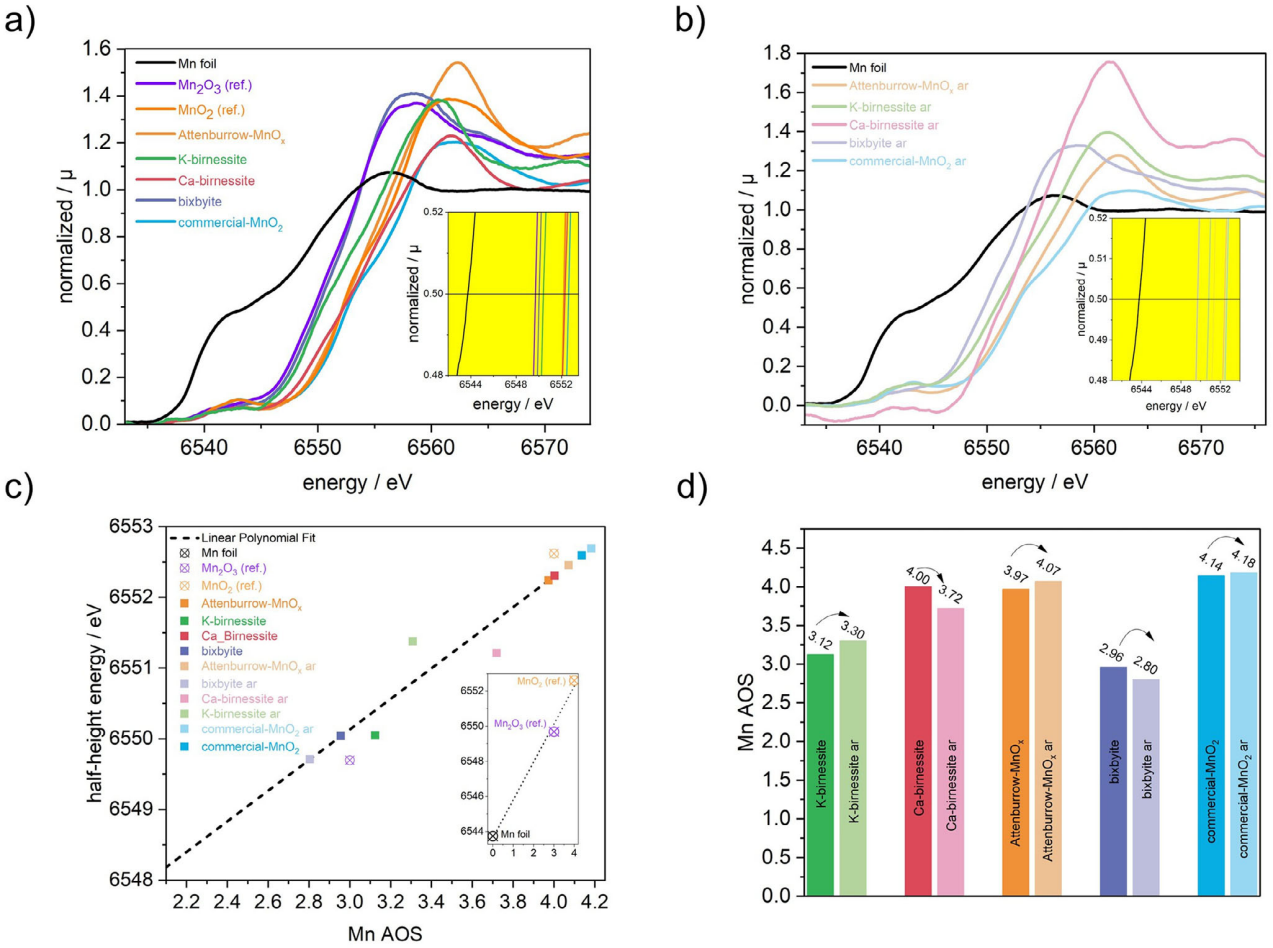
Mn K‐edge XANES data of the MnO_x_ series and its correlation with Mn average oxidation states (AOS). (a) Normalized XANES spectra of the pristine oxides and reference materials. (b) Normalized XANES spectra of the corresponding post‐reaction samples (“ar”) together with Mn foil. All spectra were background‐subtracted and normalized to a unit edge step; energies were aligned to a simultaneously measured Mn foil. The yellow windows in (a) and (b) show the edge region used to extract the half‐height energy E_1/2_. (c) Half‐height energy E_1/2_ plotted versus Mn AOS. Filled symbols denote pristine samples and reference compounds; open symbols denote post‐reaction samples. The dashed line represents the linear calibration used to convert E_1/2_ values to Mn AOS. (d) Mn AOS values derived from the XANES analysis before and after the reactions of MnO_x_ with *trans*‐pent‐2‐en‐1‐ol.

In a more detailed analysis, the XANES spectra of the MnO_x_ after reaction with the allylic alcohol show subtle but diagnostic changes (Figure 7). Most MnO_x_ exhibit only small changes in the absorption edge position, indicating that the average Mn oxidation state is largely retained within the accuracy of the method. For Ca‐birnessite ar, an average Mn oxidation state of approximately +3.7 after reaction was detected by both redox titration and XANES analysis, indicating moderate Mn reduction compared with pristine Ca‐birnessite. The bixbyite (Mn_2_O_3_) spectrum shifts slightly to lower XANES energy (average Mn valence ∼3.0 to ∼2.8), indicating a minor reduction of Mn^3+^ to Mn^2+^ consistent with its low but non‐zero activity. By contrast, K‐birnessite shows a slight upward edge shift (∼3.1–3.3 in the half‐height analysis). Given the small magnitude, this trend lies close to the method accuracy (∼±0.1–0.2 in AOS), and we refrain from a firm mechanistic interpretation. A possible scenario is that work‐up selectively removes a small, more reduced Mn fraction, leaving the recovered solid marginally enriched in higher‐valent Mn. However, we have no independent evidence for Mn dissolution, and ion chromatography shows only minor differences in Mn content (see ESI, Table S2). Within the same accuracy limits, the Attenburrow‐MnO_x_ and commercial MnO_2_ change their Mn oxidation states only slightly, consistent with the modest EXAFS variations. Overall, the XANES‐derived AOS values match the structural interpretation of the EXAFS data described above.

### 2.4 Influence of MnO_x_ Surface Areas

It is well known that large surface areas of solid reagents are highly beneficial in heterogeneous reactions like the one studied here. We therefore carried out nitrogen physisorption experiments at liquid nitrogen temperature and analyzed the isotherms (see ESI, Figure S14) by the BET model for the surface area and DFT fitting to obtain the pore size distribution. The *S*_BET_ surface areas determined in this way are listed in Table 2 and vary largely from bixbyite with the smallest (11 m^2^/g) to Ca‐birnessite with the largest *S*_BET_ value of 255 m^2^/g. However, when looking at the trends found for the reactivity in the alcohol oxidation reaction, it is clear that there is no clear correlation between the S_BET_’s of the MnO_x_ and *trans*‐pent‐2‐en‐1‐ol conversion. The Attenburrow‐MnO_x_ was clearly the most active material (∼100% conversion), but only features an intermediate surface area of 97 m^2^/g. The commercial MnO_2_ and Ca‐birnessite have very different *S*_BET_ (91 vs. 255 m^2^/g, respectively), but identical conversion rates of ∼70%. A good correlation between low reactivity and low surface areas is only found for bixbyite and K‐birnessite, both oxides with *S*_BET_ ≤50 m^2^/g (see ESI, Figures S14–S16). Since the length of the *trans*‐pent‐2‐en‐1‐ol molecule can be calculated as about 1 nm [31] based on its bond lengths, the micropores play a particularly important role. However, as can be seen in the pore size distribution plot (see ESI, Figure S17 and S18), the contribution of the micropores can be neglected. The mesopores are also relevant for the accessibility of the molecule, as they contribute significantly to the available surface area. The pore size distribution of all catalysts shows a maximum for pore diameters between 6nm and 7 nm. Among the materials studied, Ca‐birnessite exhibits the highest fraction of mesopores, while bixbyite shows the lowest micro‐ and mesoporosity. Additionally, the pore size distribution shows a rather narrow mesopore size distribution for both Ca‐Birnessite and the commercial MnO_2_, while the fraction of larger mesopores increases for Attenburrow‐MnO_2_ and K‐Birnessite. It thus seems to us that only the combination of oxidation state, structure and surface area can provide a meaningful overall picture explaining the strongly differing final yields of *trans*‐pent‐2‐en‐1‐al observed in our study.

### 2.5 Cinnamyl Alcohol as Alternative Substrate

The entire investigation presented so far focused on the reaction of the manganese oxides with a single substrate, *trans*‐pent‐2‐en‐1‐ol. In order to probe whether the reactivity trends described above might also be valid for other substrates, the already known oxidation of *trans*‐3‐phenylprop‐2‐en‐1‐ol (cinnamyl alcohol) was investigated, again using the same set of the five different MnOx as possible oxidation agents (Schemes 1 and 3). Also, for this reaction, ^1^H NMR spectroscopy proved to be an excellent tool to monitor the reaction progress and to determine product yields (see ESI, Figure S21 for details).

**SCHEME 3:**
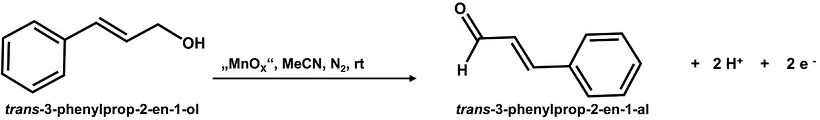
Oxidation of *trans*‐3‐phenylprop‐2‐en‐1‐ol (cinnamyl alcohol) to *trans*‐3‐phenylprop‐2‐en‐1‐al (cinnamaldehyde) using manganese oxides.

As in the case of *trans*‐pent‐2‐en‐1‐ol described above, we again observed a superb selectivity of the reaction with the corresponding allylic aldehyde *trans*‐3‐phenylprop‐2‐en‐1‐ol (cinnamaldehyde) being the only detectable oxidation product in all cases. And indeed, we observe a nearly identical ranking of substrate conversions after 1 h of reaction time (Figure 8), with the Attenburrow‐MnO_X_ achieving a yield of ∼100%, Ca‐birnessite and the commercial MnO_2_ closely behind with 76% and 77%, respectively, and only very low conversions for K‐birnessite (8 %) and bixbyite (1 %). The slightly higher yields of Ca‐birnessite and the commercial MnO_2_ in comparison to *trans*‐pent‐2‐en‐1‐ol may be explained by the extended π‐system of cinnamyl alcohol facilitating the oxidation process. But overall, the similarity between the reaction rates for the two substrates is remarkable and might indicate that Figure 8 really illustrates a general reactivity trend at least for the oxidation of allylic alcohols by MnO_x_.

**FIGURE 8:**
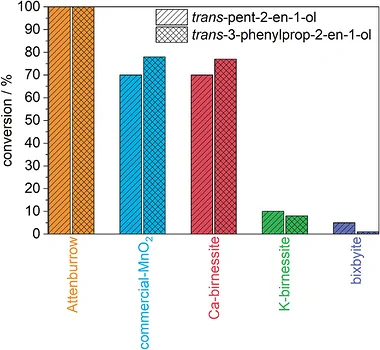
Comparison of the conversion yields of *trans*‐pent‐2‐en‐1‐ol and *trans*‐3‐phenylprop‐2‐en‐1‐ol to their respective aldehydes by the five manganese oxides investigated in this study. Reaction conditions: Mn:substrate ratio 10:1, reaction time 60 min., solvent acetonitrile.

## 3 Conclusion

Our detailed investigation of the well‐known selective oxidation of allylic alcohols (here mainly modelled by *trans*‐pent‐2‐en‐1‐ol) to aldehydes by manganese oxides clearly revealed the importance of both MnO_x_ structure and Mn oxidation state for this transformation. As indicated especially by our pXRD and XAS data, MnO_x_ materials of low crystallinity and average Mn oxidation states close to +4.0 are especially well suited for this reaction and show high substrate conversions. The three best‐performing oxides of this study (Attenburrow‐MnO_x_, Ca‐birnessite, and the commercial “activated MnO_2_”) can all be categorized as belonging to the birnessite family of layered MnO_x_ and combine low crystallinity with Mn oxidation states well above +3. All three also contain a substantial number of secondary cations, which obviously tune their reactivities as well as for example, the comparison between the Ca‐birnessite (very active) and the K‐birnessite (moderately active) illustrates. An additional series of oxidation reactions, this time with cinnamyl alcohol as substrate, revealed an identical trend in MnO_x_ reactivities. As always layered oxides with secondary cations are the best‐performing oxidation agents, a label like “M'MnO_x_” should in our view be more correctly used in reaction schemes for the oxidation of allylic alcohols by manganese oxides, as the descriptor “MnO_2_” is clearly too simplistic to describe the oxidation agent.

Some additional results from our study point at possibilities to use and improve the oxidation of allylic alcohols by M'MnO_x_ in the future: (1) large substrate‐accessible specific surface areas of the oxides proved to be of significant (though not crucial) importance for reactivity, and especially an enhancement of S_BET_ for the very well‐performing Attenburrow‐MnO_x_ from its currently rather low value of ∼100 m^2^/g could therefore be beneficial; (2) as the synthetic procedures for Ca‐birnessite and the Attenburrow‐MnO_x_ are surprisingly similar (see experimental section), it might be possible to prepare a “Ca‐variant” of the Attenburrow‐MnO_x_ with probably even better reactivity; (3) it would be interesting to investigate more of the oxidation processes shown in Scheme 1 (especially those involving substrates other than allylic alcohols) to probe the “generality” of the reactivity trend shown in Figure 8; and (4) as Mn^4+^ ⇆ Mn^3+^ redox state changes seem to be a common feature of the active oxides (as also known for MnO_x_‐based water‐oxidation catalysts), it might be possible to also carry out the oxidation of allylic alcohols electrocatalytically. The latter would truly represent a new twist for this classic of organic synthesis.

## 4 Experimental Section

### 4.1 General Considerations

All reagents and solvents were purchased from commercial sources and used without further purification or pretreatment. Deionized water from a Veolia flex 4 water purification system (electrical resistivity>18 MΩ·cm^−1^ at 25°C) was used for all experiments.

### 4.2 Experimental Details and Analytical Procedures

***Powder X‐ray diffraction (XRD)***: Powder X‐ray diffraction patterns of the pulverized samples were recorded at room temperature on a Bruker D8 Discover diffractometer in Bragg‐Brentano geometry (goniometer radius 280 mm) with Cu K_α_ radiation (*λ*  =  154.187 pm), variable divergence slit at the primary beam side and a LYNXEYE XE‐T detector. Data collection was carried out for the 2*θ* range of 7°–100° with a step size of 0.26° and a time of 0.5 s per step. Data evaluation and phase analysis were performed using the Bruker DIFFRAC.EVA software.

***X‐ray absorption spectroscopy (XAS/EXAFS)***: X‐ray absorption spectroscopy (XAS) measurements were performed in transmission mode using an EasyXAFS300+ spectrometer equipped with a KETEK AXAS‐M silicon drift detector (SDD) and an Ag X‐ray tube as the source. Measurements were performed at the Mn K‐edge for both the samples and their reference materials at ambient conditions. Standard XAS data processing procedures were applied using the ATHENA [28] software, including normalization, preedge background subtraction and post‐edge absorption edge calibration. The k^2^‐weighted extended X‐ray absorption fine structure (EXAFS) spectra, k^2^χ(k), were extracted in the range Δ*k* ≈ 3–11 Å^−^^1^. The resulting spectra were Fourier transformed to R‐space, yielding FT|k^2^χ(k)| in the interval Δ*R* ≈ 0‐6 Å. Quantitative EXAFS fitting was performed using the Larch software package, which integrates theoretical phase and amplitude functions generated by FEFF8. For this study, theoretical models were constructed using crystallographic input files of the reference structure, allowing accurate simulation of scattering paths for manganese atoms and their coordination shells. The fits were refined by adjusting structural parameters such as bond distances, coordination numbers and Debye–Waller factors to best match the experimental data. To further explore the local structural environment, wavelet transform (WT) analysis was performed on the Mn K‐edge EXAFS spectra. The analysis utilized a Morlet mother wavelet function with a frequency parameter (*η*) of 5 and a Gaussian envelope width (*σ*) of 2, optimizing resolution in both k‐ and R‐space. This approach allowed clear differentiation of overlapping contributions from multiple scattering paths within the R range of 1–4 Å, providing a more intuitive two‐dimensional visualization of EXAFS features in momentum and real space.

***ATR–FTIR spectroscopy***: All IR spectra were measured at room temperature before and after reaction of the manganese oxides using a Thermo Fisher Scientific Nicolet iS10 spectrometer equipped with a ZnSe ATR‐unit. The shown spectra are the result of 64 individual spectra.

***Redox titrations***: For the titration, a sodium oxalate solution (0.2 m, 7.5 mL) and sulfuric acid (0.5 m, 10 mL) were added to approx. 100 mg of carefully weighed manganese oxide. The mixture was heated to 70°C and then titrated with potassium permanganate solution (0.02 m) until conversion. The oxidation states of the manganese were then calculated based on the Mn content of the oxide determined by ion chromatography (see below).

***Nuclear magnetic resonance (NMR)***: ^1^H NMR spectra were recorded on Bruker Avance Neo 400, Bruker Avance III HD 300 and Bruker DPX 200 spectrometers. The proton chemical shifts are given relative to TMS. The proton spectra were calibrated using the solvent signal as a secondary standard. The integration procedure is described in detail in Section 1 of the ESI.

***Ion chromatography***: Approx. 10 mg of the respective manganese oxide was carefully weighed and then dissolved in 1 mL of a 9:1 mixture of HNO_3_ (65%) and H_2_O_2_ (30%). The sample was then diluted to 250 mL and analyzed using a Metrohm 882 Compact IC plus ion chromatograph with a Metrohm Metrisep C4 cation column (100 mm). A solution of HNO_3_ (2 m, 0.85 mL) and 1,6‐pyridinedicarboxylic acid (0.02 m, 35 mL) diluted to 1 L using deionized water served as the eluent. The data was analyzed using Metrohm's MagICNet software.

***N_2_‐physisorption***: N_2_‐physisorption measurements were performed on a *Micro300* from *3P Instruments GmbH* and were conducted at 77 K using a fixed dose amount sorption control. The sample mass was determined by weighing the glass cell alone before degassing and then again with the sample after the degassing procedure (60 min to 200°C, hold 6 h). The obtained isotherms were analysed using the *Quantachrome ASiQwin* (Version 5.21). The multi‐point BET surface area was determined in the relative pressure range (p/p_0_) in the linear regimes of the Rouquerol and the BET plots. The pore size distribution and cumulative pore volume were determined using a N_2_ at 77 K on silica (cylindr. pores, NLDFT adsorption branch) fitting model in the *p*/*p*_0_ range of 0.001–1.0.

### 4.3 Syntheses of Manganese Oxides

The syntheses of the four not commercially available manganese oxides were carried out from common manganese starting materials following published routes (Scheme 4).

**SCHEME 4:**
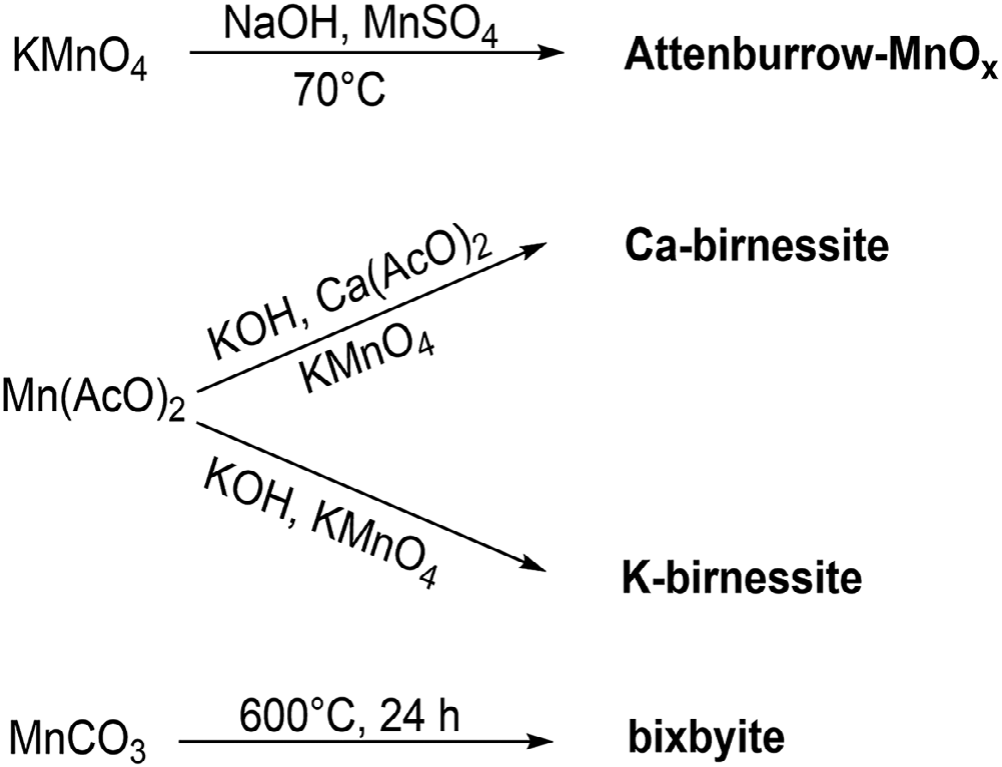
Synthesis routes for the manganese oxides prepared for this study.

**Synthesis of Attenburrow‐MnO_2_**: Analogous to the procedure described by Attenburrow et al. [15], a solution of MnSO_4_·H_2_O (90.0 g, 53.2 mmol) in H_2_O (160 mL) and a solution of NaOH (71.6 g, 107 mmol) in H_2_O (107 mL) were added simultaneously within 1 h to a hot and vigorously stirred solution of KMnO_4_ (103 g, 650 mmol) in H_2_O (640 mL) kept at 70°C. After stirring the reaction mixture for an additional 2 h at room temperature, the precipitate was filtered off, washed with H_2_O until the filtrate was colorless, and the brown product was dried for 48 h in air at 100°C (yield: 95 g, ∼65 %).

**Synthesis of Ca‐birnessite**: Following a synthesis developed by Suib et al. [32], and modified by us [5], a solution of Mn(AcO)_2_·4H_2_O (98.0 g, 400 mmol) and Ca(AcO)_2_·H_2_O (21.2 g, 120.0 mmol) in H_2_O (640 mL) was added dropwise to a vigorously stirred solution of KOH (180 g, 6.40 mol) in H_2_O (640 mL) to give a brownish suspension. A solution of KMnO_4_ (23.8 g, 150.0 mmol) in H_2_O (2.4 L) was then added dropwise within 45 min and the reaction mixture was then stirred for another 2 h. The precipitate was filtered off, washed with H_2_O until the filtrate was colorless, and the crude product was dried for 60 h in air (yield: 84 g, ∼90%).

**Synthesis of K‐birnessite**: K‐birnessite was synthesized similar to the Ca‐birnessite by adding a solution of Mn(AcO)_2_·4H_2_O (92.0 g, 400 mmol) in H_2_O (370 mL) to a stirred solution of KOH (252 g, 5.0 mol) in H_2_O (370 mL) within 30 min followed by dropwise addition of KMnO_4_ (23.8 g, 150.0 mmol) in H_2_O (1.20 L). The precipitate was filtered off, washed with H_2_O until the filtrate was colorless, and the crude product was dried for 24 h in air at 60°C (yield: 63 g, ∼90%).

**Synthesis of bixbyite**: For the synthesis of bixbyite, MnCO_3_ (80.0 g, 701 mmol) was heated to 600°C for 24 h in air in a muffle furnace [5]. The black product was used without further purification steps (yield: 70 g, ∼100%).

### 4.4 General Procedure for the Oxidation of Allylic Alcohols

All reactions were carried out at room temperature. The chosen amount of the respective manganese oxide was purged with nitrogen for 30 minutes before the addition of the alcohol (10 mmol) in N_2_‐saturated acetonitrile (50 mL). Samples were taken periodically from the stirred reaction mixture to observe the reaction by NMR. After complete conversion of the alcohol (120 min), the manganese oxide was carefully filtered off, washed with acetonitrile and dried at 100°C in air in a drying cabinet. For the special case of Ca‐birnessite, we found that this procedure had a profound influence on the oxide structure, as a conversion of the material to hausmannite was detected (see ESI, Figure S19). However, when Ca‐birnessite was heated to 100°C without first reacting it with the substrate, this was not observed and so we concluded that only the combination of the reaction with the alcohol and subsequent heating leads to this transformation. In consequence, Ca‐birnessite was dried at room temperature for post‐reaction analyses.

## Conflicts of Interest

The authors declare no conflicts of interest.

## Supporting information




**Supporting File 1**: chem71376‐sup‐0001‐SuppMat.pdf

## Data Availability

The data that support the findings of this study are available from the corresponding author upon reasonable request.
