# Supplementary material for: Oxidation of Allylic Alcohols by Manganese Oxides: Elucidating the Influences of MnOx Structure and Mn Oxidation State on Reactivity
Source: Chemistry. 2026 Jul 20;32(39):e71376. doi: 10.1002/chem.71376 (PMC13715479; doi:10.1002/chem.71376)
Supplement: Supplementary file 1 [file CHEM-32-e71376-s001.pdf]

Supporting Information

Oxidation of allylic alcohols by manganese oxides: elucidating the influences of MnO_x structure and Mn oxidation state on reactivity

Erdogan Kasisari[a], Leandra Meyer[a], S. Esmael Balaghi* [a-c], Harald Scherer[a], Patrick Elsässer[a-d], Anna Fischer*[a-d], Philipp Kurz*[a][b]

[a] E. Kasisari, L. Meyer, Dr. S. E. Balaghi, Dr. H. Scherer, P. Elsässer, Prof. A. Fischer, Prof. Ph. Kurz
Institut für Anorganische und Analytische Chemie
Albert-Ludwigs-Universität Freiburg
Albertstraße 21, 79104 Freiburg, Germany
E-mail: esmael.balaghi@fit.uni-freiburg.de; philipp.kurz@ac.uni-freiburg.de

[b] Freiburger Materialforschungszentrum (FMF)
Albert-Ludwigs-Universität Freiburg
Stefan-Meier-Straße 21, 79104 Freiburg, Germany

[c] Freiburg Center for Interactive Materials and Bioinspired Technologies (FIT)
Georges-Köhler-Allee 105, 79110 Freiburg, Germany

[d] Cluster of Excellence Living, Adaptive, and Energy-autonomous Materials Systems (livMatS)
Georges-Köhler-Allee 105, 79110 Freiburg, Germany

TABLE OF CONTENTS

1. NMR evaluation of the reaction control 3

2. Influence of water...8

3. Comparison of pentenol, pentenal and Ca-birnessite after the reaction............9

4. Ca-Birnessite EXAFS Fit: Parameters & Results 10

5. N_2-Physisorption 12

6. Ion chromatography 15

7. The effect of post-treatment on Ca-birnessite 16

8. NMR evaluation of cinnamonalcohol..18

1. NMR evaluation of the reaction control

For the conversion analysis, each reaction tracing was analysed by NMR at different times. Two NMR spectra (Attenburrow-MnO_X (Fig. S 2) after 0 min and Attenburrow-MnO_X (Fig. S 4) after 60 min) are shown below as examples to assign the NMR signals of the reactant (*trans*-pent-2-en-1-ol, Fig. S 1) and product (*trans*-pent-2-en-1-al, Fig. S 3). All subsequent NMRs were baseline corrected by hand using a polynomial fit.

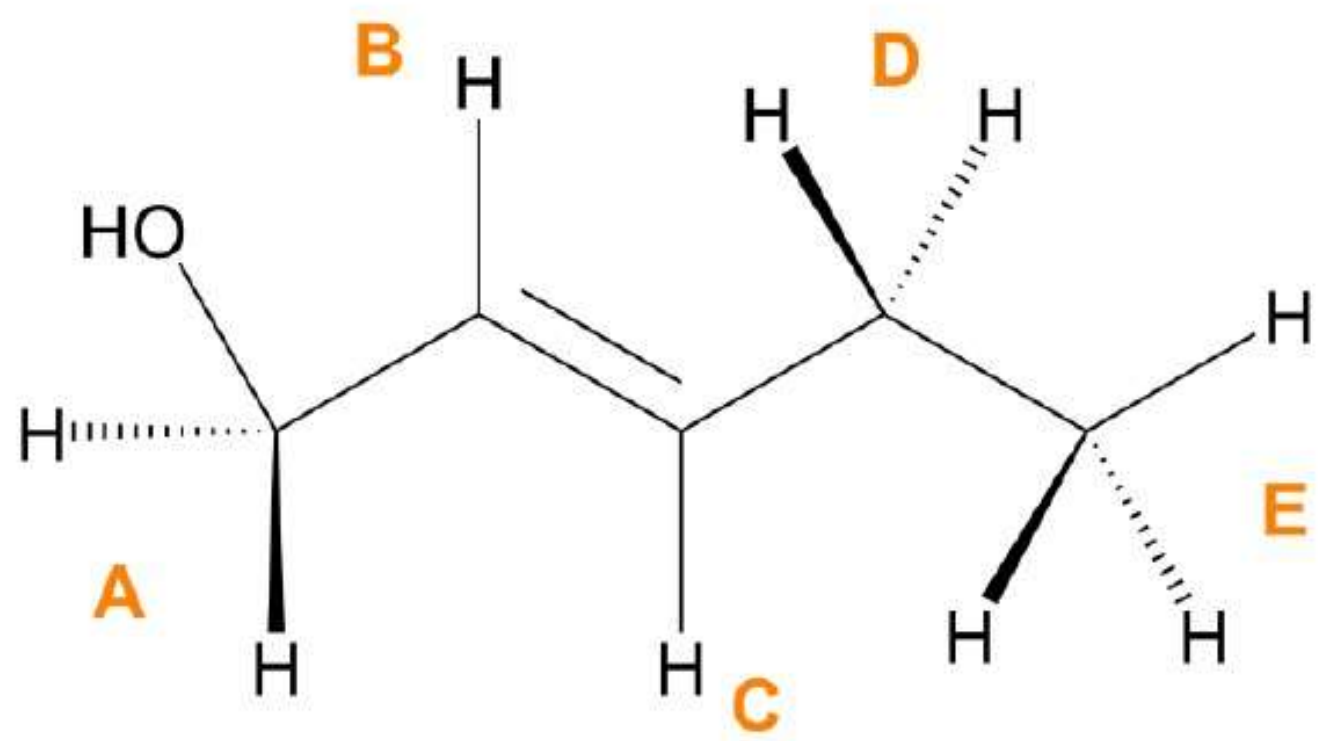

Figure S 1. Structural formula of *trans*-pent-2-en-1-ol with assignment of the NMR-relevant groups.

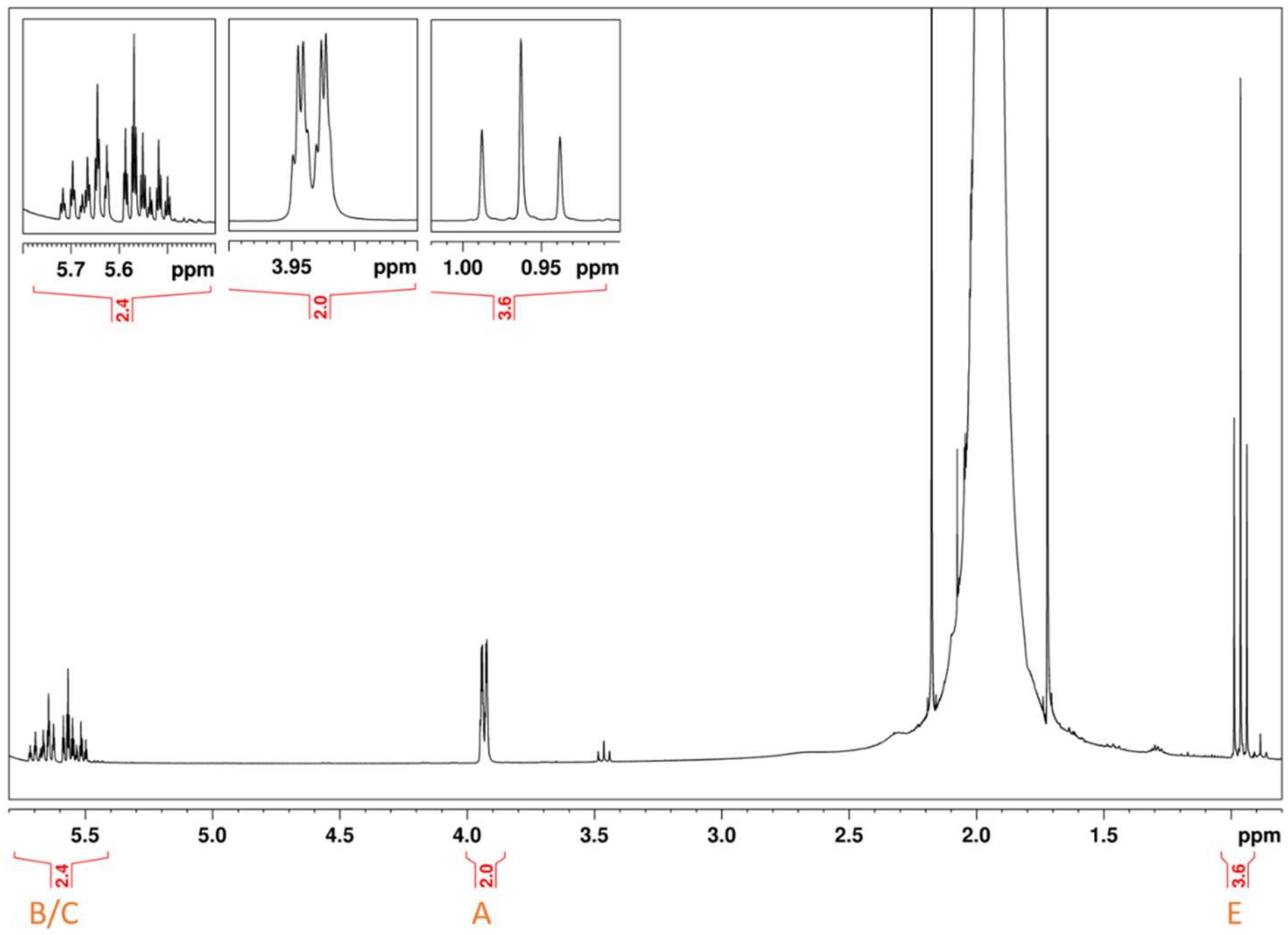

Figure S 2. ^{1}H NMR spectrum of the reaction mixture at the start in acetonitrile with Attenburrow-MnO_X as oxidant with a frequency of 300.18 MHz at 25 °C.

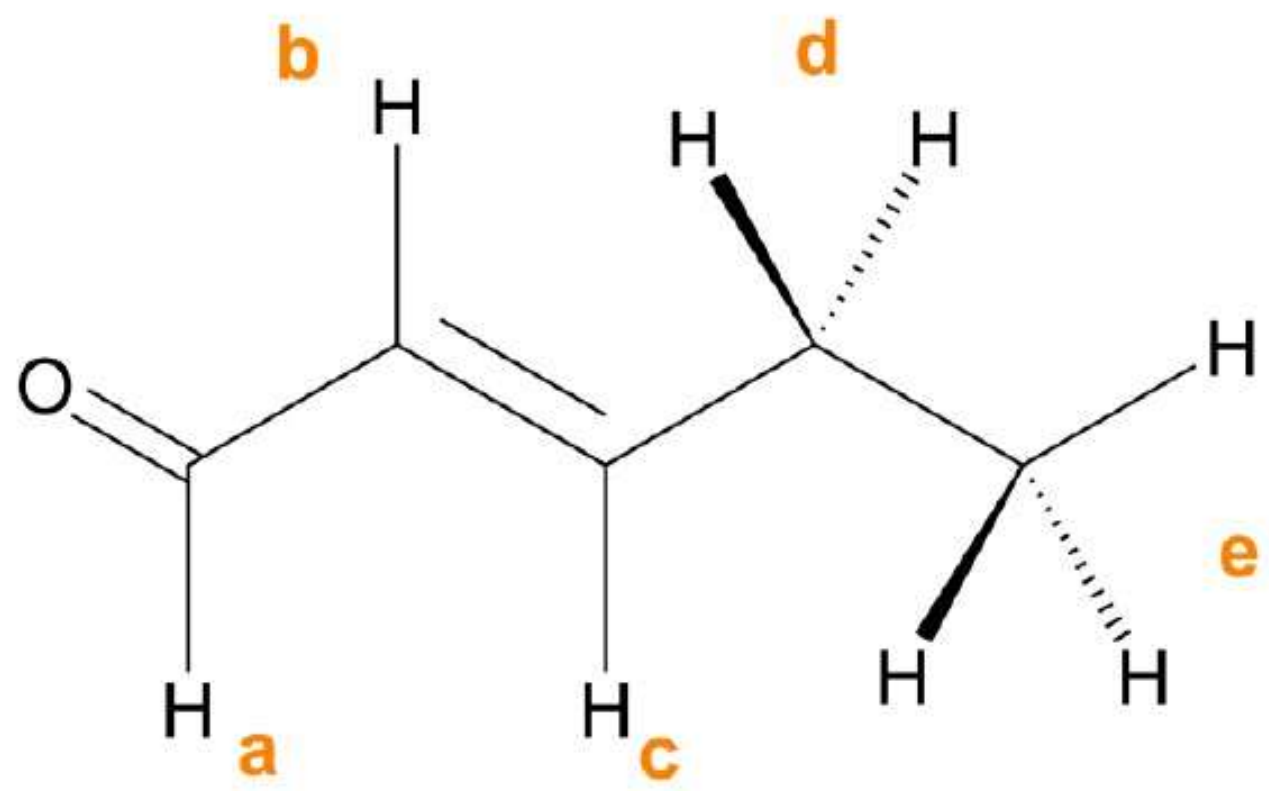

Figure S 3: Structural formula of *trans*-pent-2-en-1-al with assignment of the NMR-relevant groups.

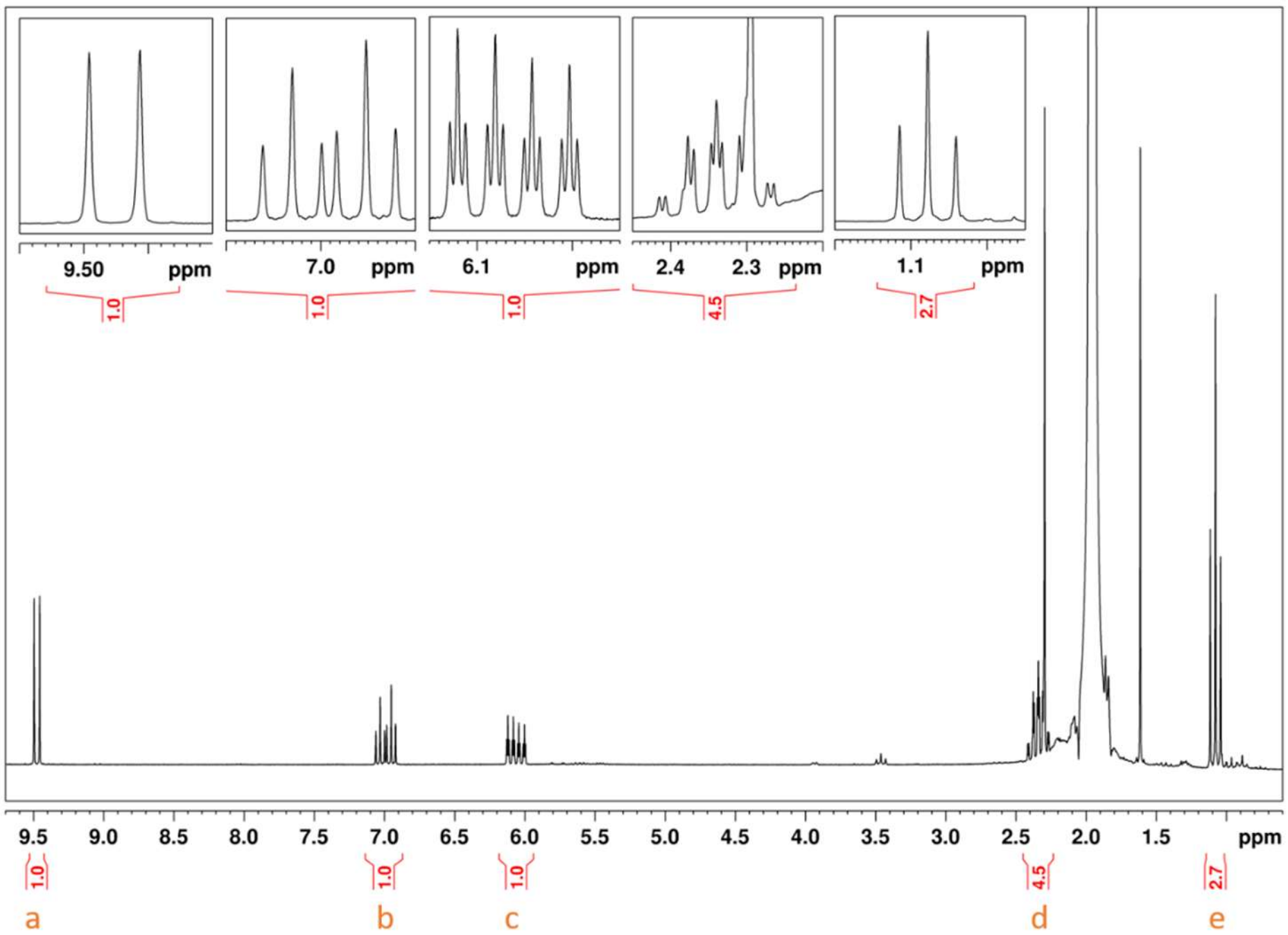

Figure S 4: ^{1}H NMR spectrum of the reaction mixture after 60 minutes in acetonitrile with Attenburrow-MnO_X as oxidant with a frequency of 200.13 MHz at 25 °C.

Signal a and signal A are used to determine the conversion, using the following equation:

$$U_P = \frac{S(a)}{S(a) + \frac{1}{2}S(A)} \cdot 100$$

The NMR spectrum of the reaction mixture of the Attenburrow-MnO_X and the substrate after 15 min reaction (Fig. S 5) can serve as an example for this quantitative analysis:

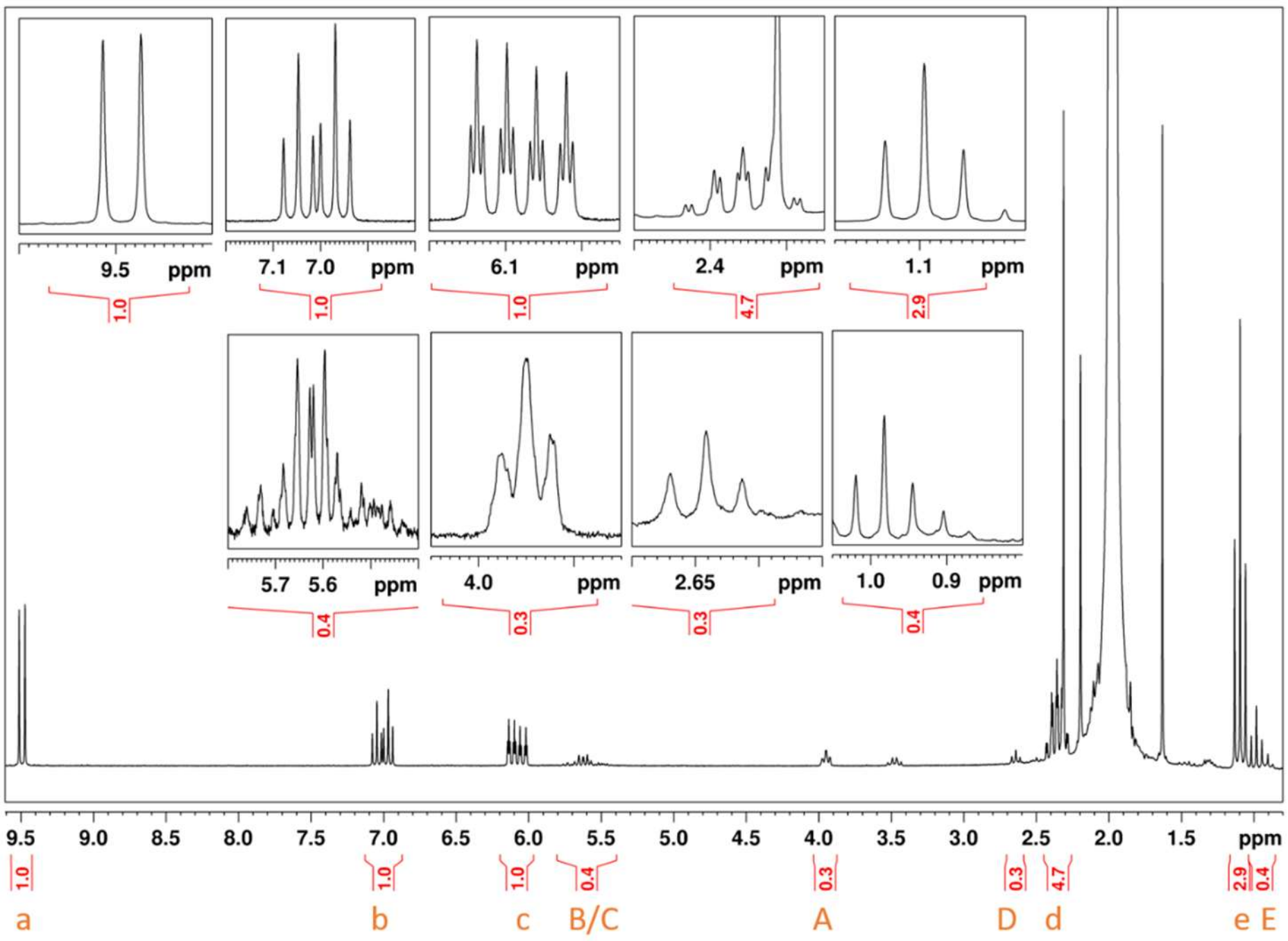

Figure S 5. ^{1}H NMR spectrum of the reaction mixture after 15 minutes in acetonitrile with the Attenburrow-MnO_X as oxidant (frequency 200.13 MHz at 25 °C).

The quantitative analysis results in:

$$U_P = \frac{1.00}{1.00 + 0.15} \cdot 100 = 86\ \%$$

In addition, the NMR spectra of the reaction mixtures with other manganese oxides at 60 min (Fig. S 6-9) are shown on the following pages.

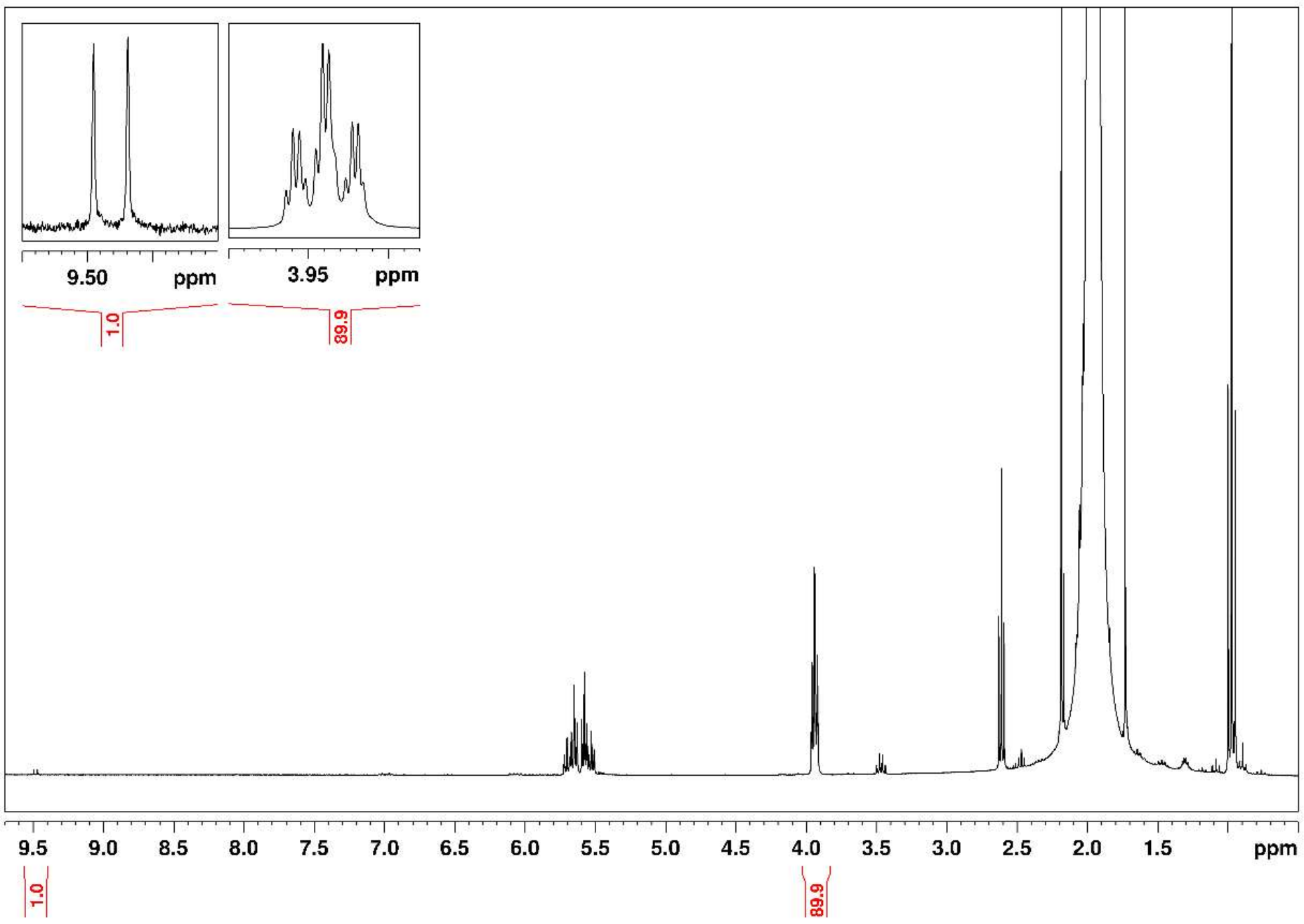

Figure S 6. ^1H NMR spectrum of the reaction mixture after 60 minutes in acetonitrile with bixbyite as oxidant with a frequency of 300.13 MHz at 25 °C.

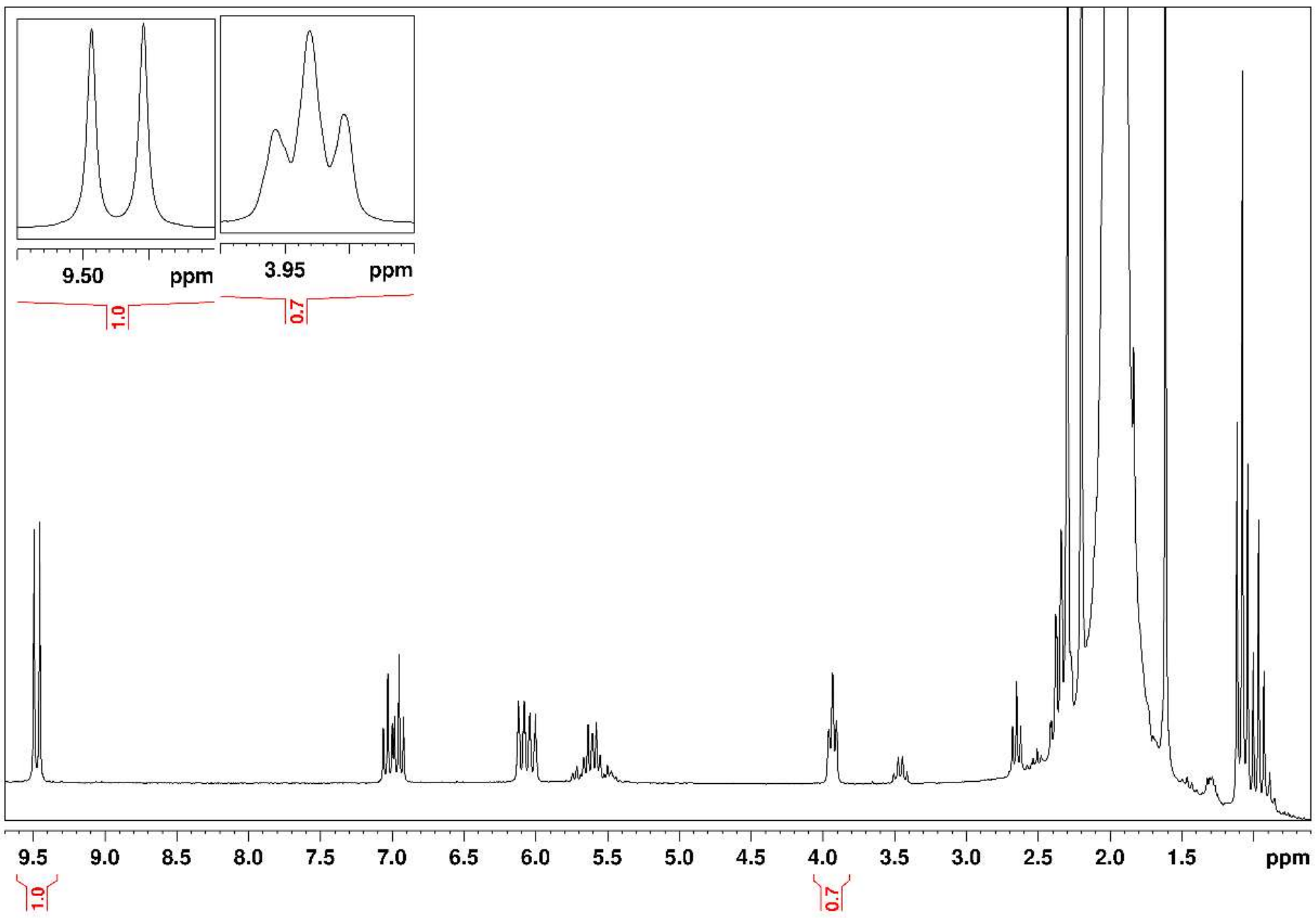

Figure S 7. ^1H NMR spectrum of the reaction mixture after 60 minutes in acetonitrile with Ca-birnessite as oxidant with a frequency of 200.13 MHz at 25 °C.

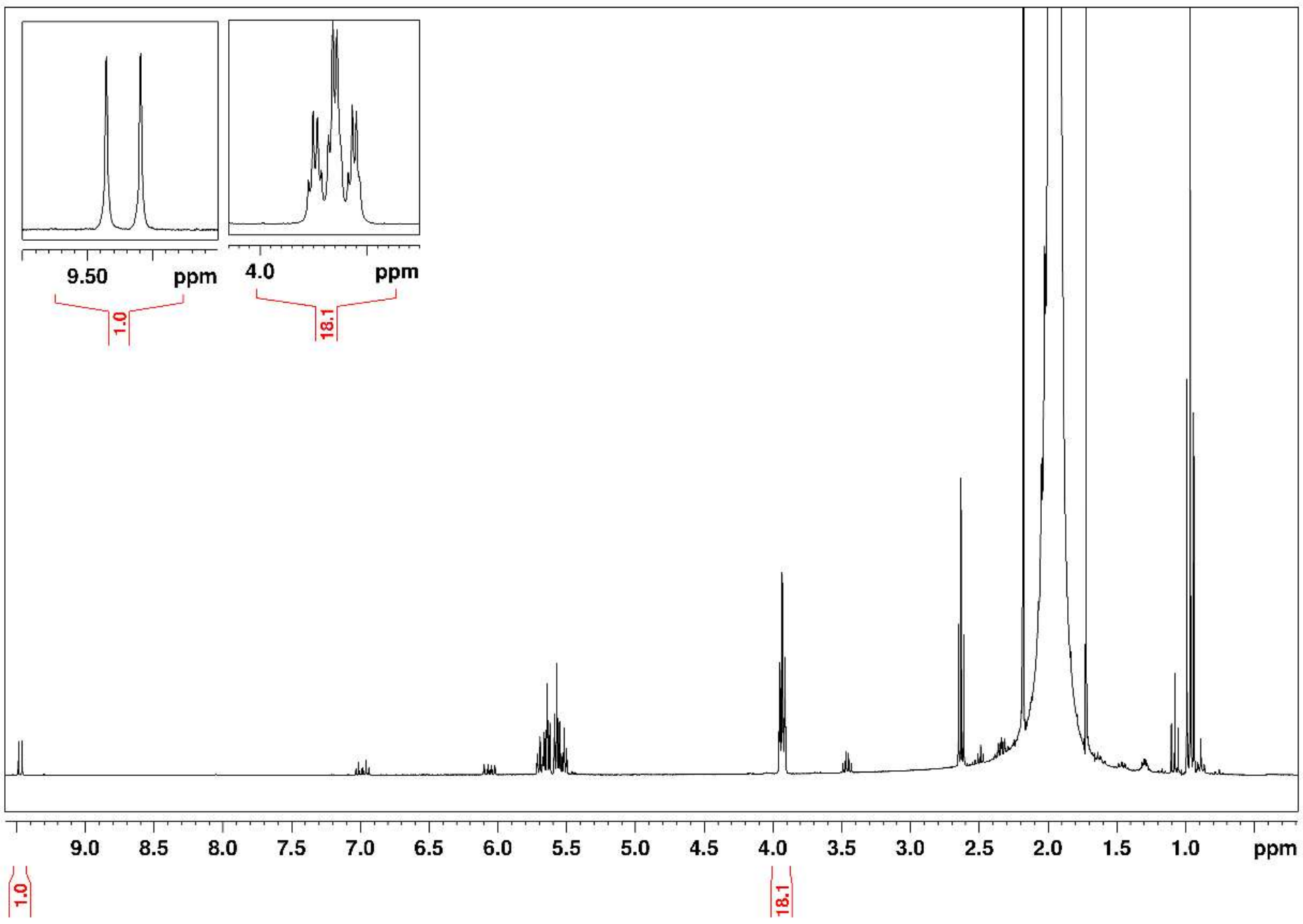

Figure S 8. ^1H NMR spectrum of the reaction mixture after 60 minutes in acetonitrile with K-birnessite as oxidant with a frequency of 300.18 MHz at 25 °C.

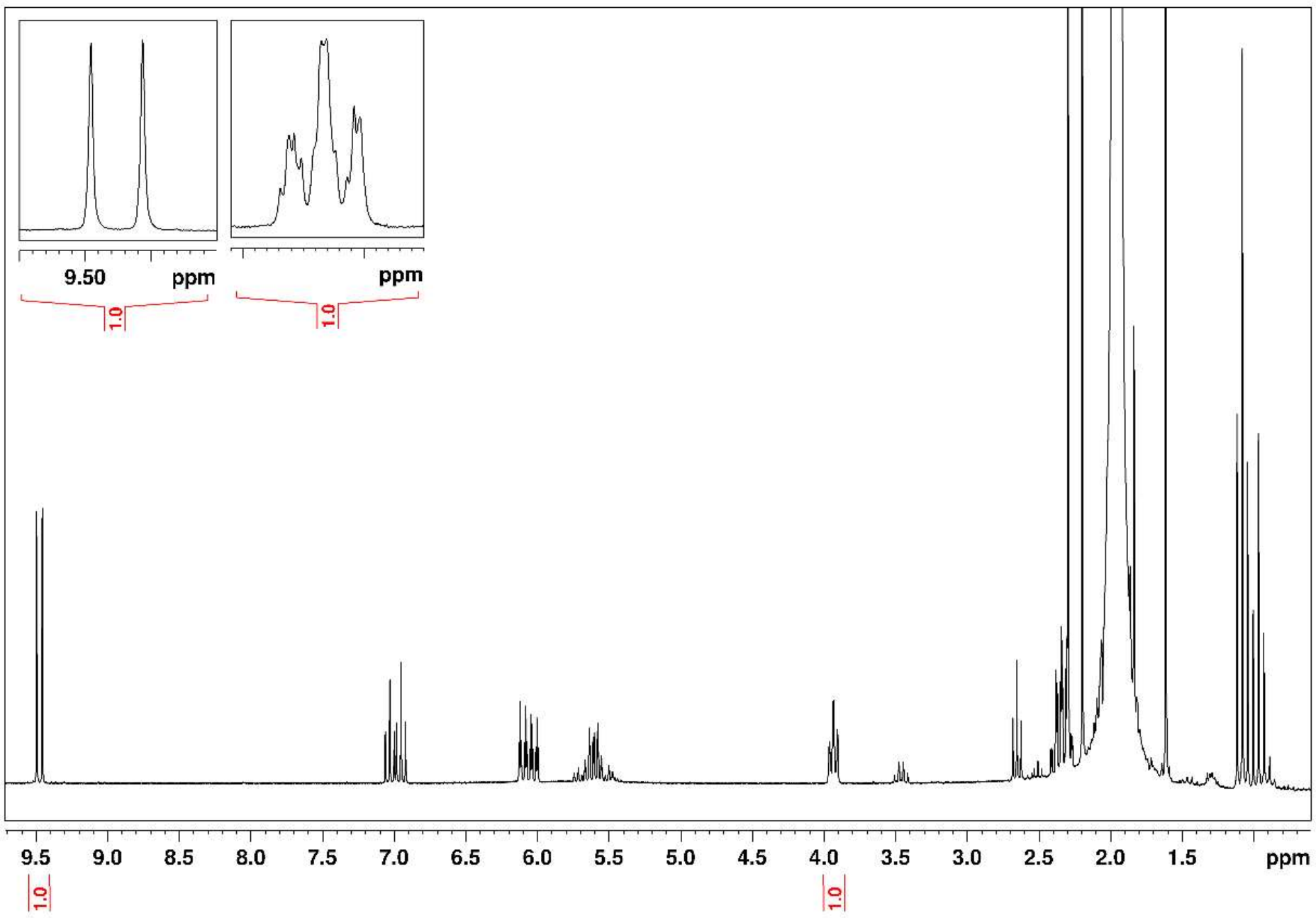

Figure S 9. ^1H NMR spectrum of the reaction mixture after 60 minutes in acetonitrile with Merck-MnO_X as oxidant with a frequency of 200.13 MHz at 25 °C.

2. Influence of water

Figure S 10 shows the conversion of the reaction with Attenburrow as oxidant under different conditions.

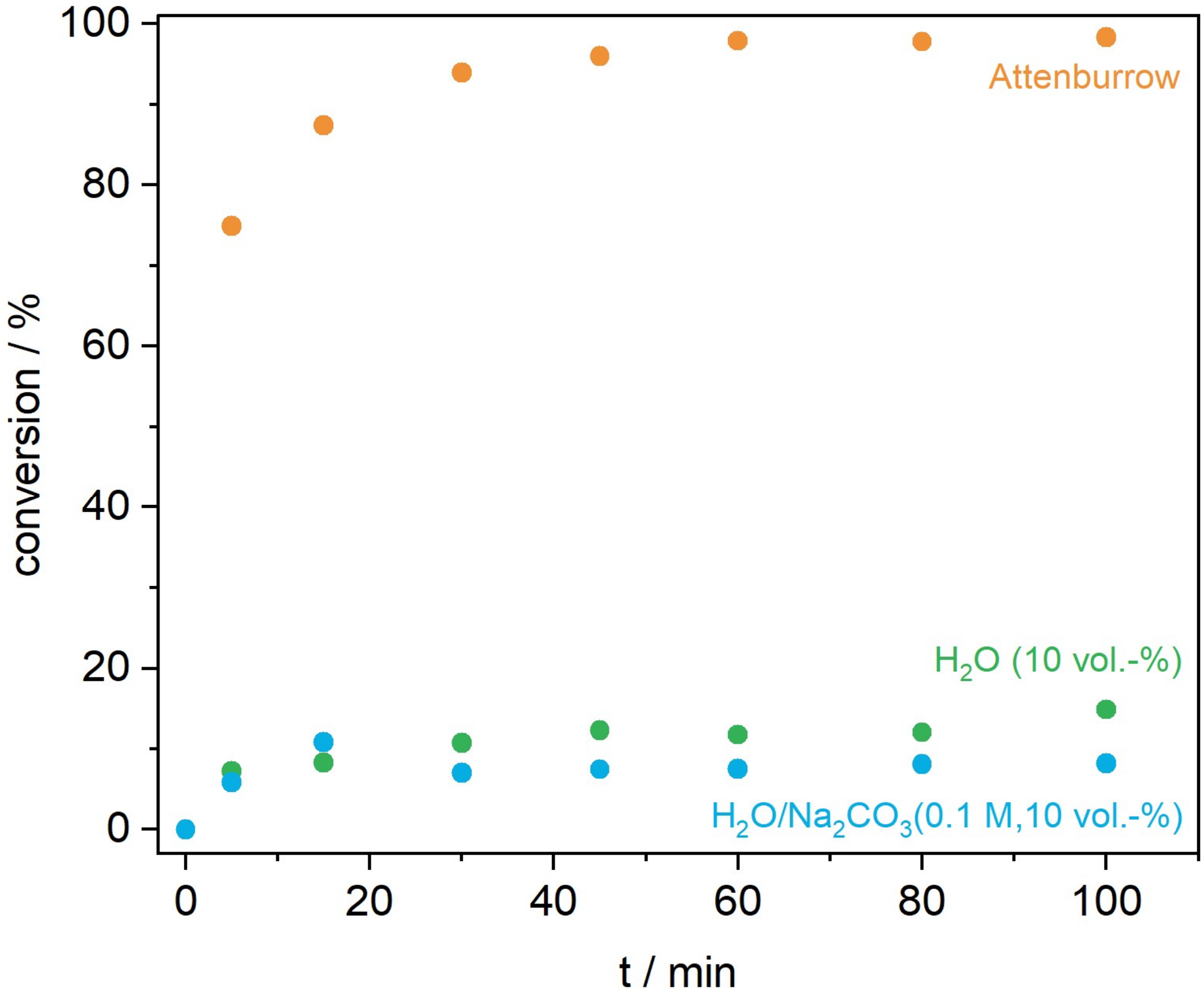

Figure S 10. Time-dependent conversion of *trans*-pent-2-en-1-ol to *trans*-pent-2-en-1-al by Attenburrow-MnO_x (Mn : substrate ratio 10 : 1) in acetonitrile without or with added water or aqueous sodium carbonate solution (0.1 M).

3. Comparison of *trans*-2-penten-1-ol, *trans*-2-penten-1-al and Ca-birnessite after the reaction

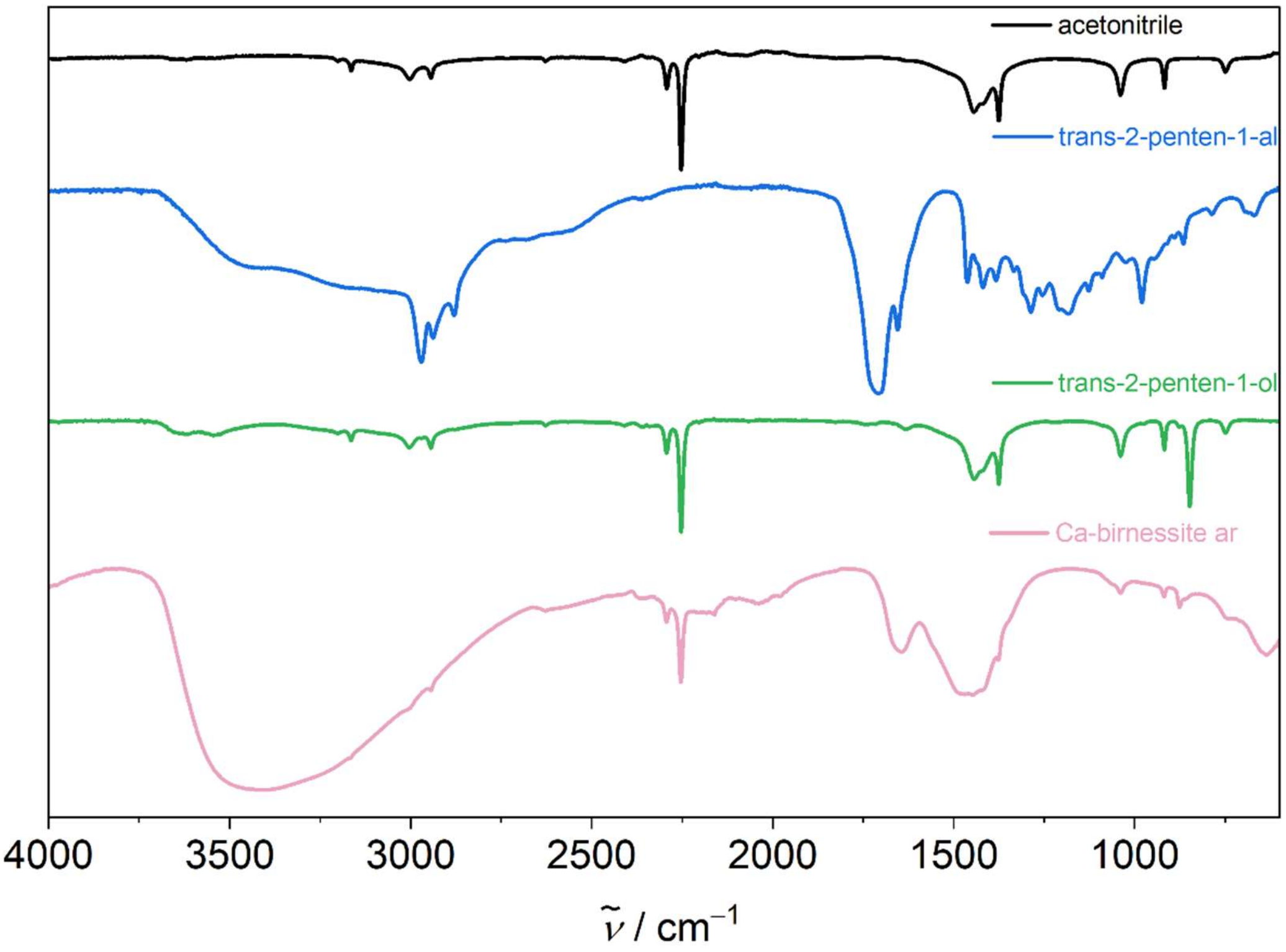

Figure S 11. Comparison of the IR spectrum for Ca-birnessite after its reaction with *trans*-2-penten-1-ol with those of the possible adsorbed species acetonitrile, *trans*-2-penten-1-ol and *trans*-2-penten-1-al.

4. Ca-Birnessite EXAFS Fit: Parameters & Results

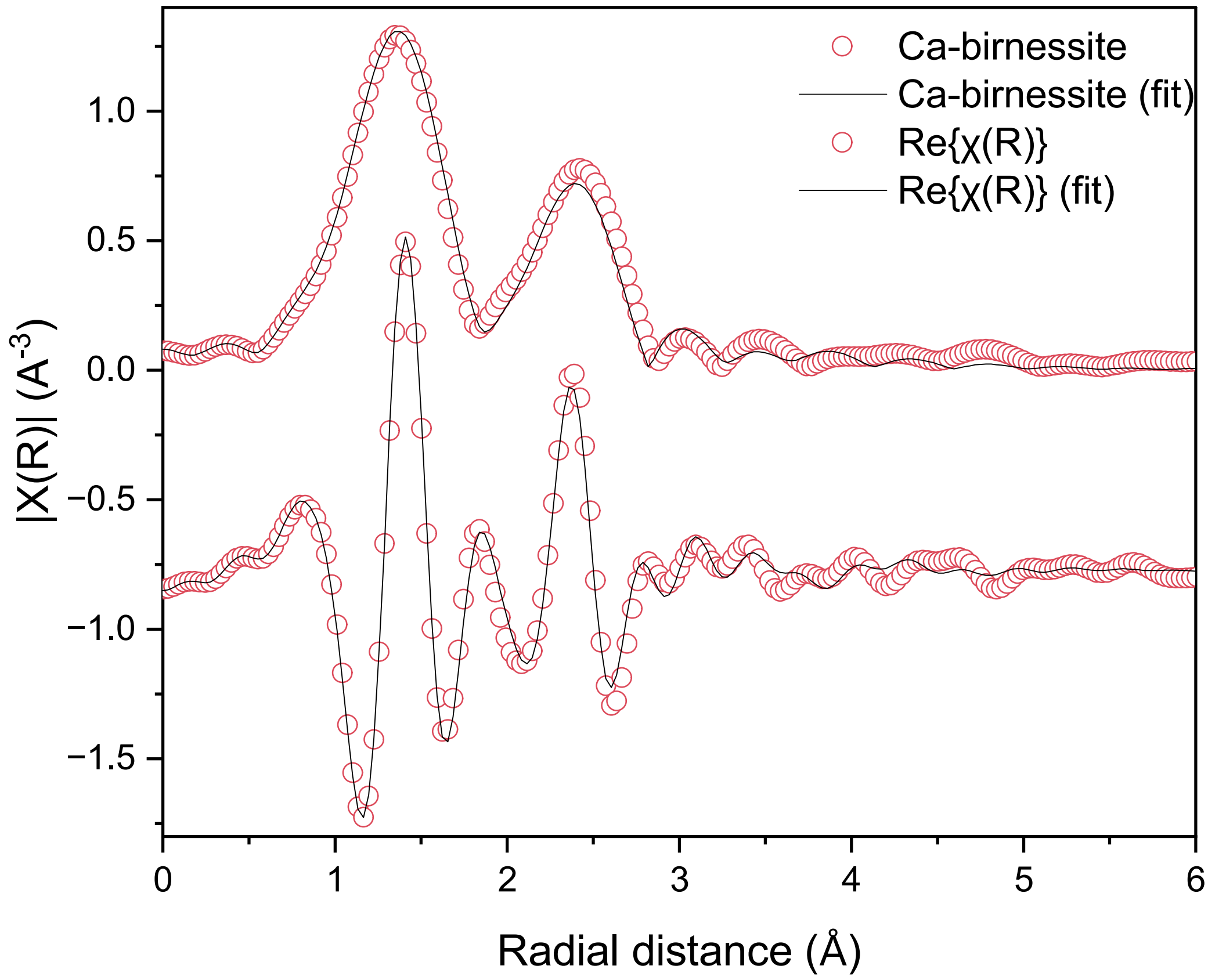

Figure S 12. Mn K-edge EXAFS of fresh Ca-birnessite in R-space. Fourier transform of k-weighted χ(k) (k = 3–11 Å^{-1}). Top: FT magnitude |χ(R)| (open circles = data; solid line = fit). Bottom: real part Re{χ(R)} (D = data, E = fit). Phase-uncorrected R is shown. The fit uses a birnessite model with two Mn–O subshells and two edge-sharing Mn–Mn paths (S_0^2 = 0.92 fixed; R-window 1.2–3.2 Å).

Table S 1. Mn K-edge EXAFS parameters for Ca-birnessite and qualitative changes after. S_0^2 = 0.92; $\Delta E_0 \approx +4.36$ eV; k = 3–11 Å^{-1}, R = 1.2–3.2 Å; R-factor ≈ 0.020.

Sample	Scatterer	CN (eff.)	R / Å	σ² / Å²	Comment
Fresh Ca-birnessite	Mn–O (short)	5.5	1.939	0.00546	Dominant octahedral Mn–O; δ-MnO_2-like
	Mn–O (long)	0.5	2.032	0.00546	Minor JT-elongated fraction (f_l ≈ 0.08)
	Mn–Mn (edge)	2	2.791	0.00869	In-plane edge-sharing
	Mn–Mn (edge)	4	2.873	0.00869	In-plane edge-sharing
Ca-birnessite after reaction					First shell largely intact; edge-sharing Mn-Mn feature attenuated and broadened, with weak additional intensity at R′ ≈ 3.1-3.3 Å (phase-uncorrected); consistent with partial loss of birnessite-type in-plane order and partial spinel/hausmannite-like reorganization.

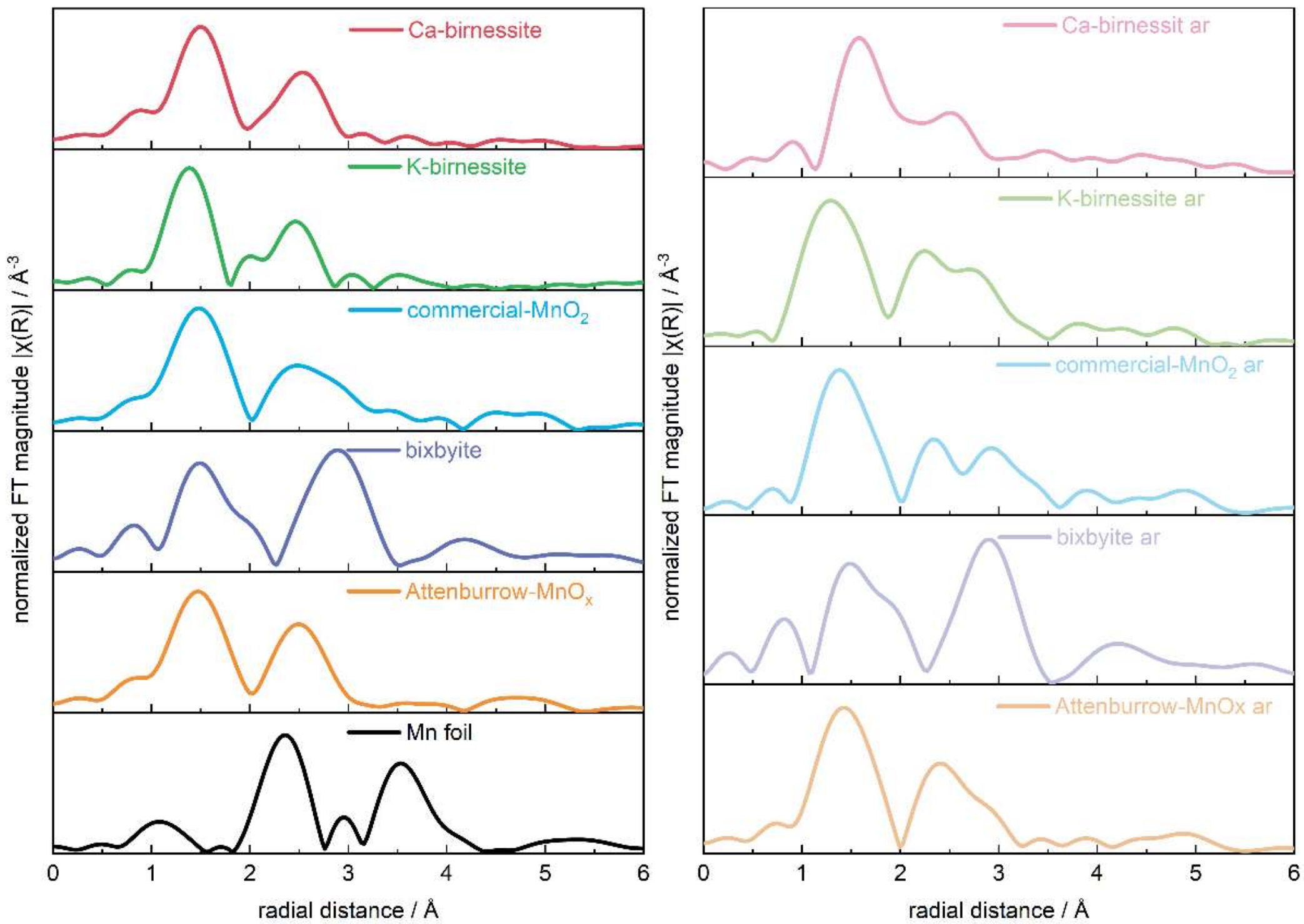

Figure S13. Mn K-edge FT-EXAFS of all oxides before reaction (left) and after reaction (right). Fourier transforms of $k^2\chi(k)$ (k = 3-11 $Å^{-1}$; Hanning window, dk = 1) are plotted as $|\chi(R)|$ versus the phase-uncorrected radial distance R′. Spectra are normalized and vertically offset for comparison. The attenuation and broadening of the edge-sharing Mn-Mn feature at R′ ≈ 2.8-3.0 Å in Ca-birnessite ar, together with additional intensity at slightly higher R′, support partial loss of birnessite-type in-plane order and partial spinel/hausmannite-like reorganization. In contrast, K-birnessite ar and commercial MnO_2 ar largely retain the edge-sharing Mn-Mn feature, while bixbyite ar retains its characteristic higher-R′ corner-sharing Mn-Mn contribution.

5. N_2-Physisorption

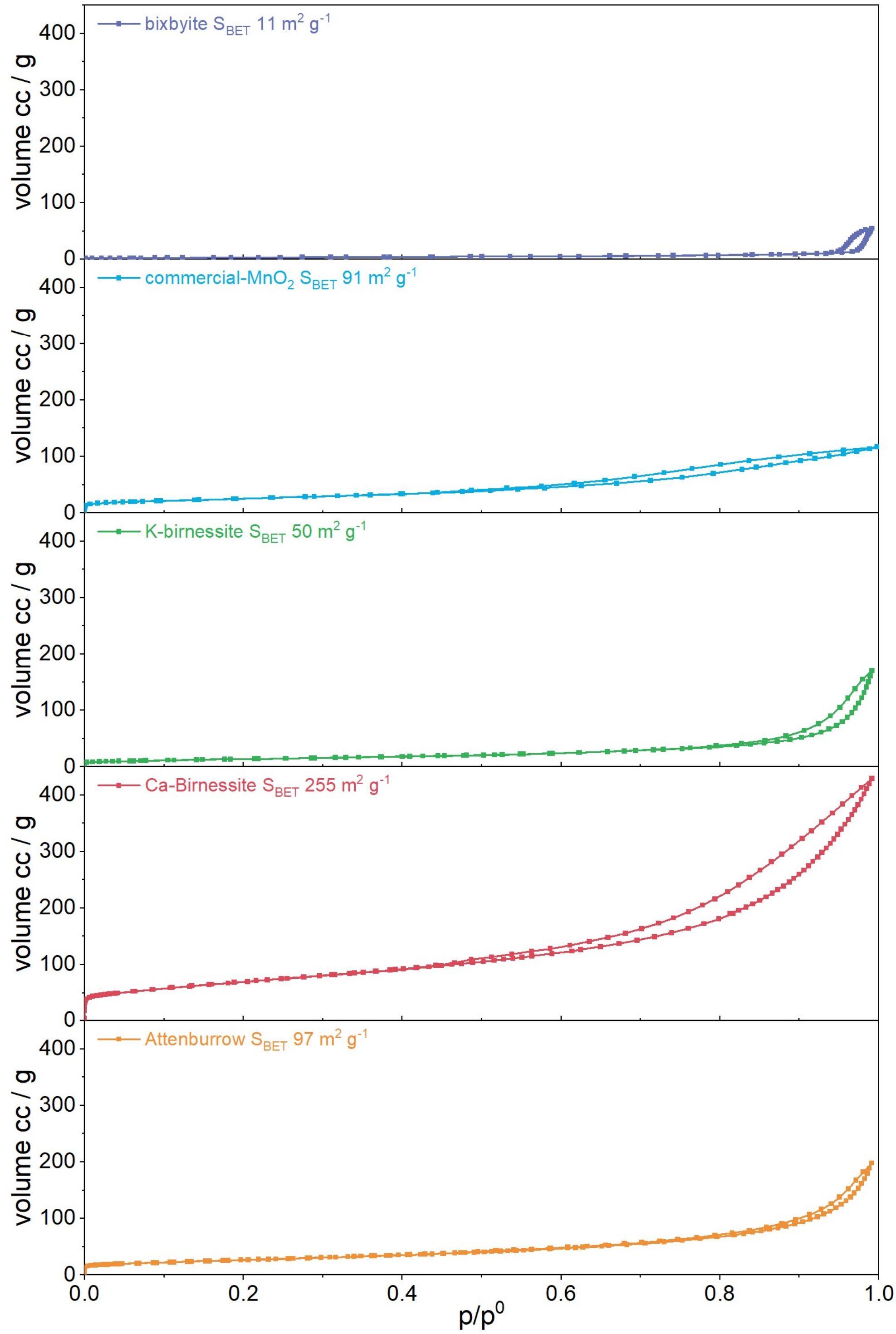

Figure S 14. Nitrogen adsorption isotherms used to determine the BET surface areas of the five manganese oxides.

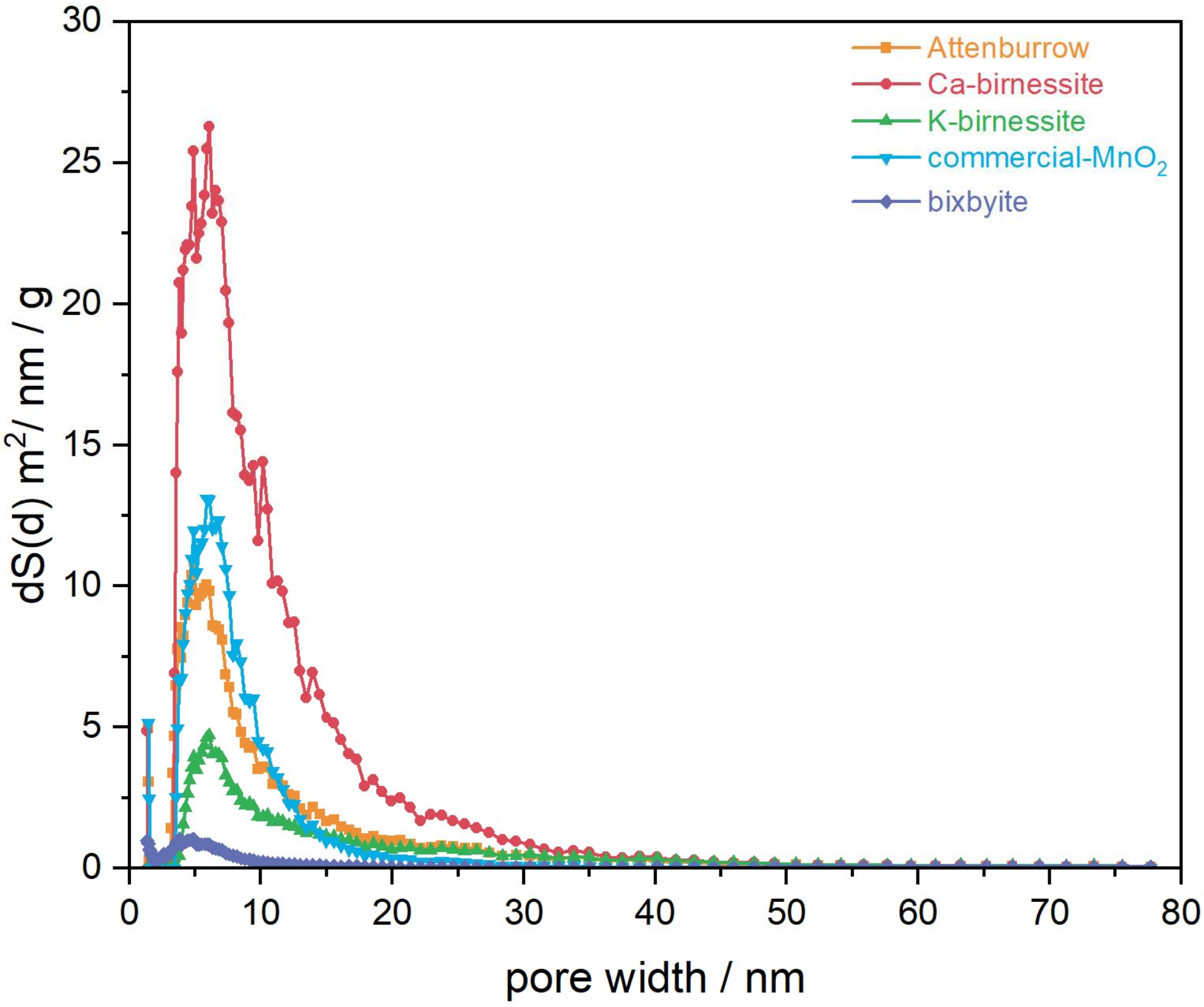

Figure S 15. Distribution of surface area as a function of pore size.

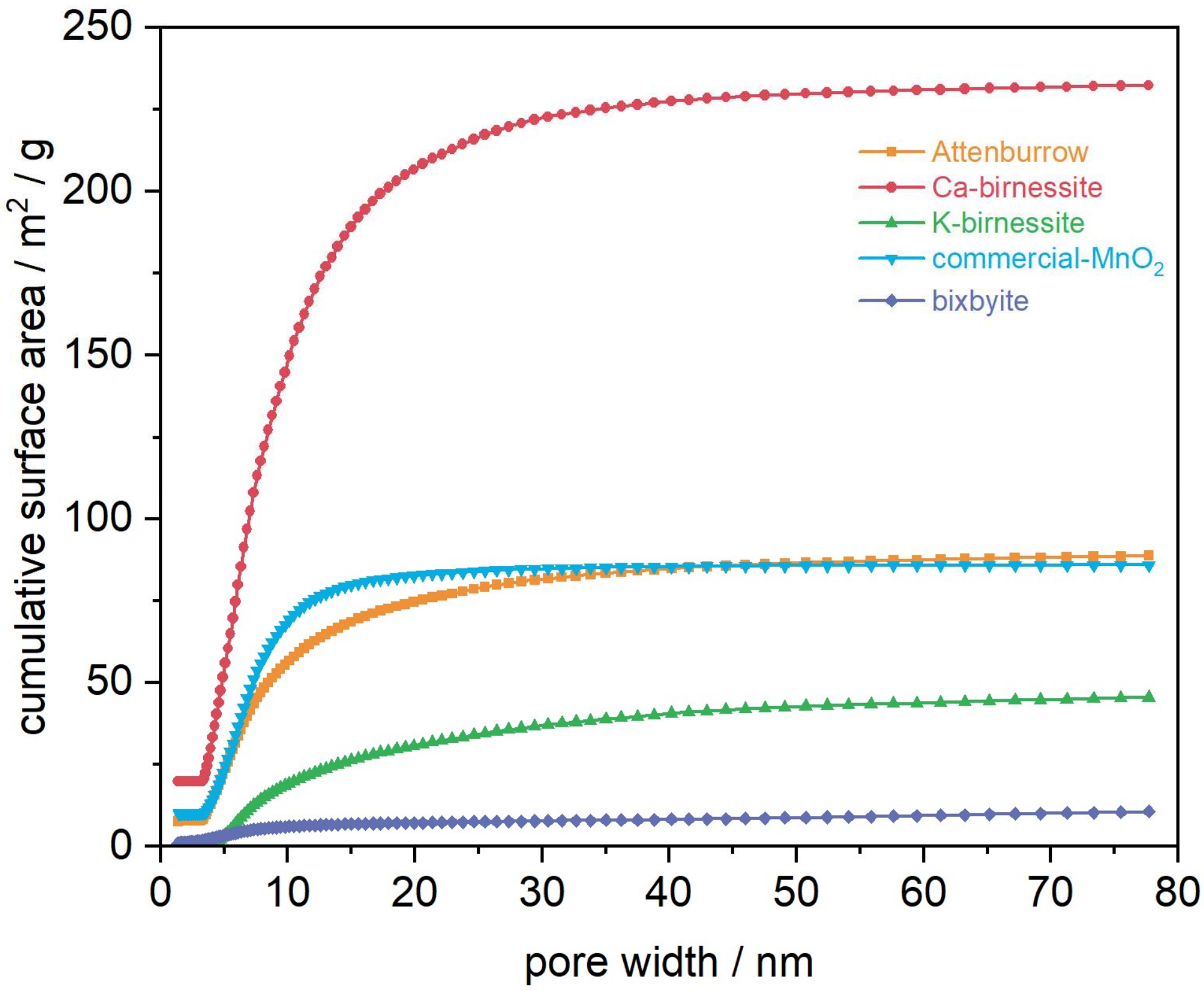

Figure S 16. Cumulative surface area as a function of pore width.

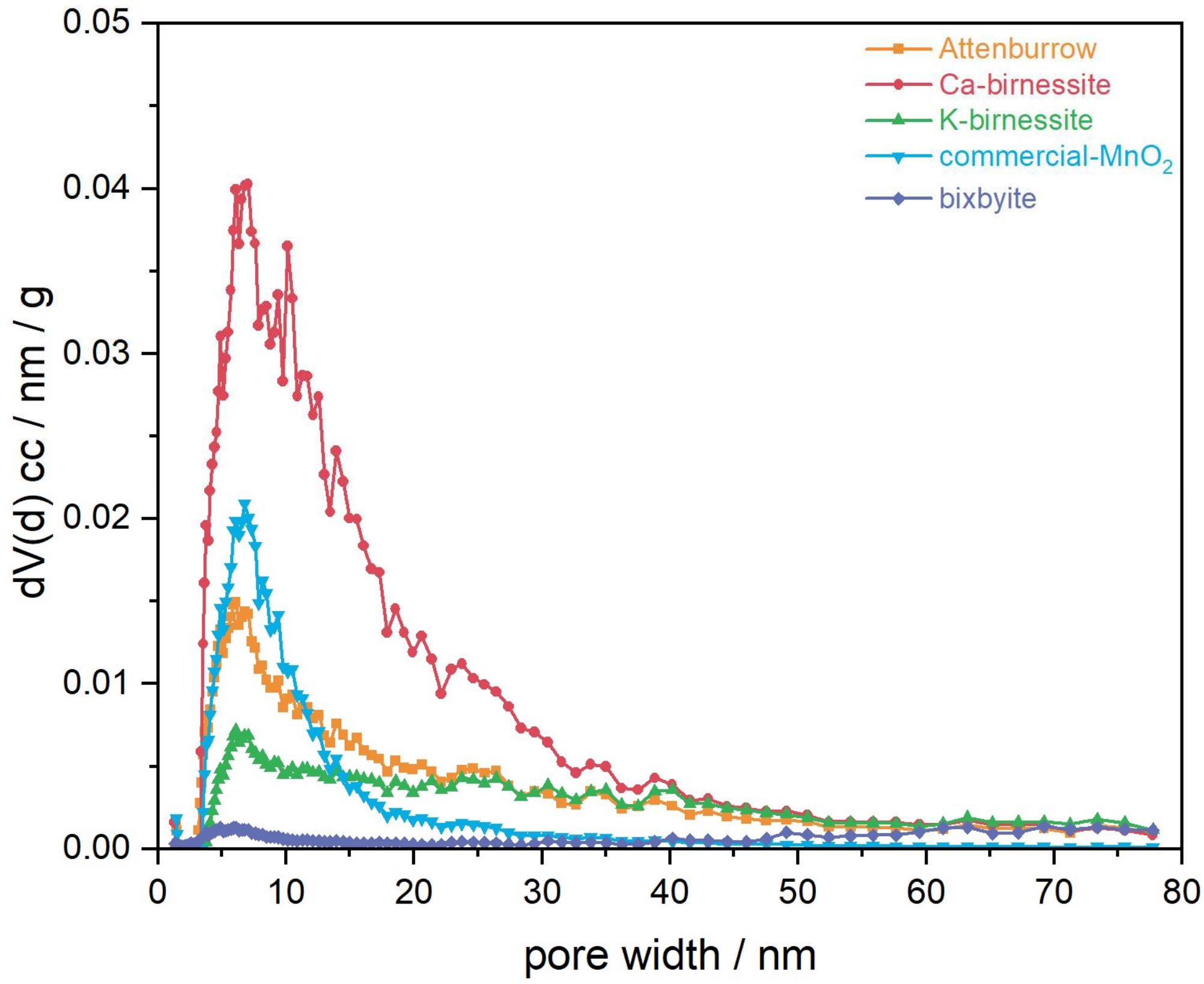

Figure S 17. Distribution of volume as a function of pore size.

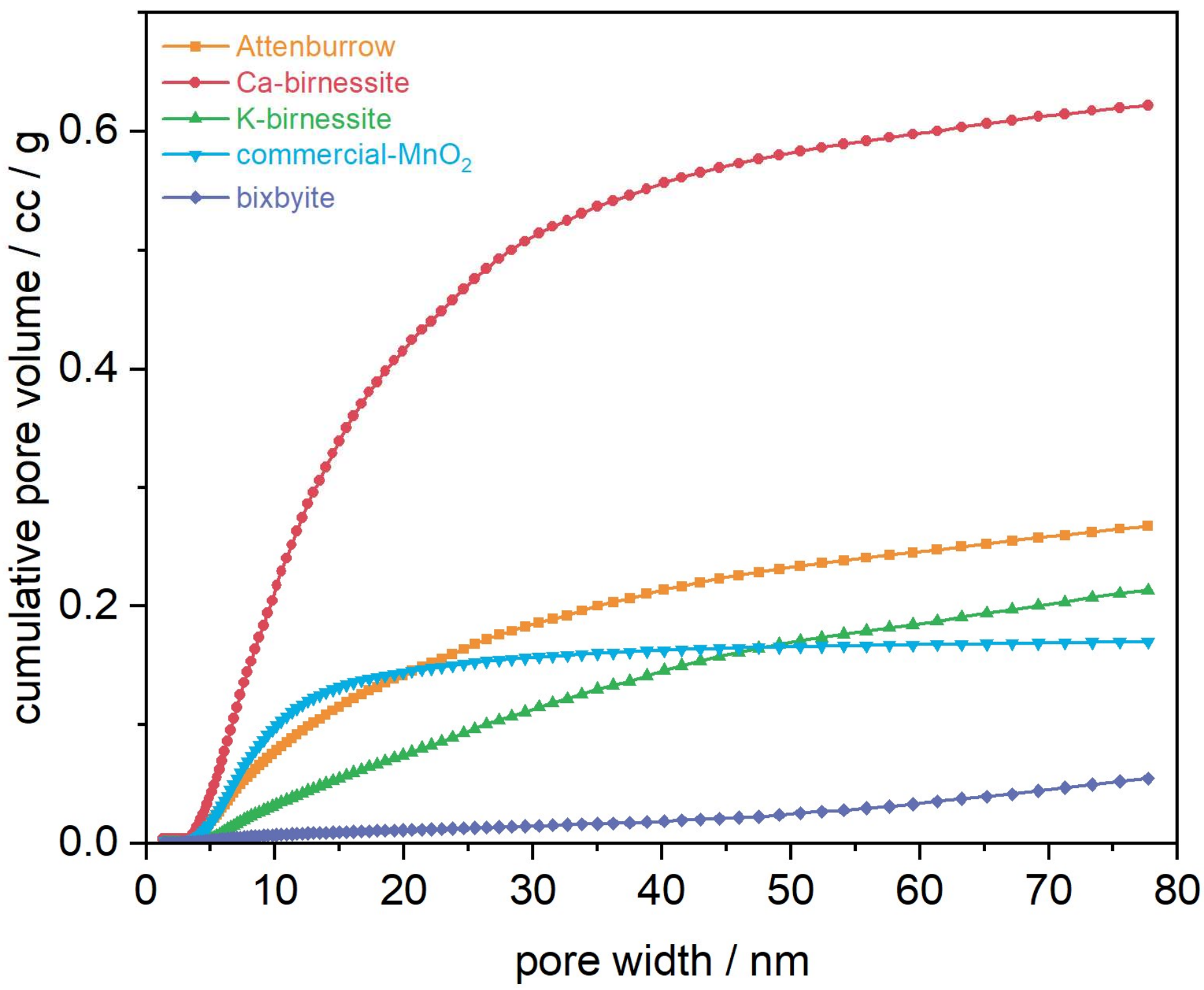

Figure S 18. Cumulative pore volume as a function of pore width.

6. Ion chromatography

Table S 2. Cation composition (w %) of the manganese oxides, determined by ion chromatography.

Sample	Manganese	Potassium	Calcium	Sodium
Attenburrow-MnO_x	54.0	4.2	0.0	5.2
Attenburrow-MnO_x ar	57.3	5.1	0.0	6.4
Ca-birnessite	42.6	5.9	6.7	0.0
Ca-birnessite ar	51.9	5.8	7.7	0.0
commercial MnO_2	62.9	0.0	2.2	0.0
commercial MnO_2 ar	62.9	0.0	0.0	0.0
K-birnessite	56.1	6.7	0.0	0.0
K-birnessite ar	56.4	7.7	0.0	0.0
bixbyite	69.2	0.0	0.0	0.0
bixbyite ar	72.7	0.0	0.0	0.0

7. Effect of the post-reaction drying temperature on Ca-birnessite

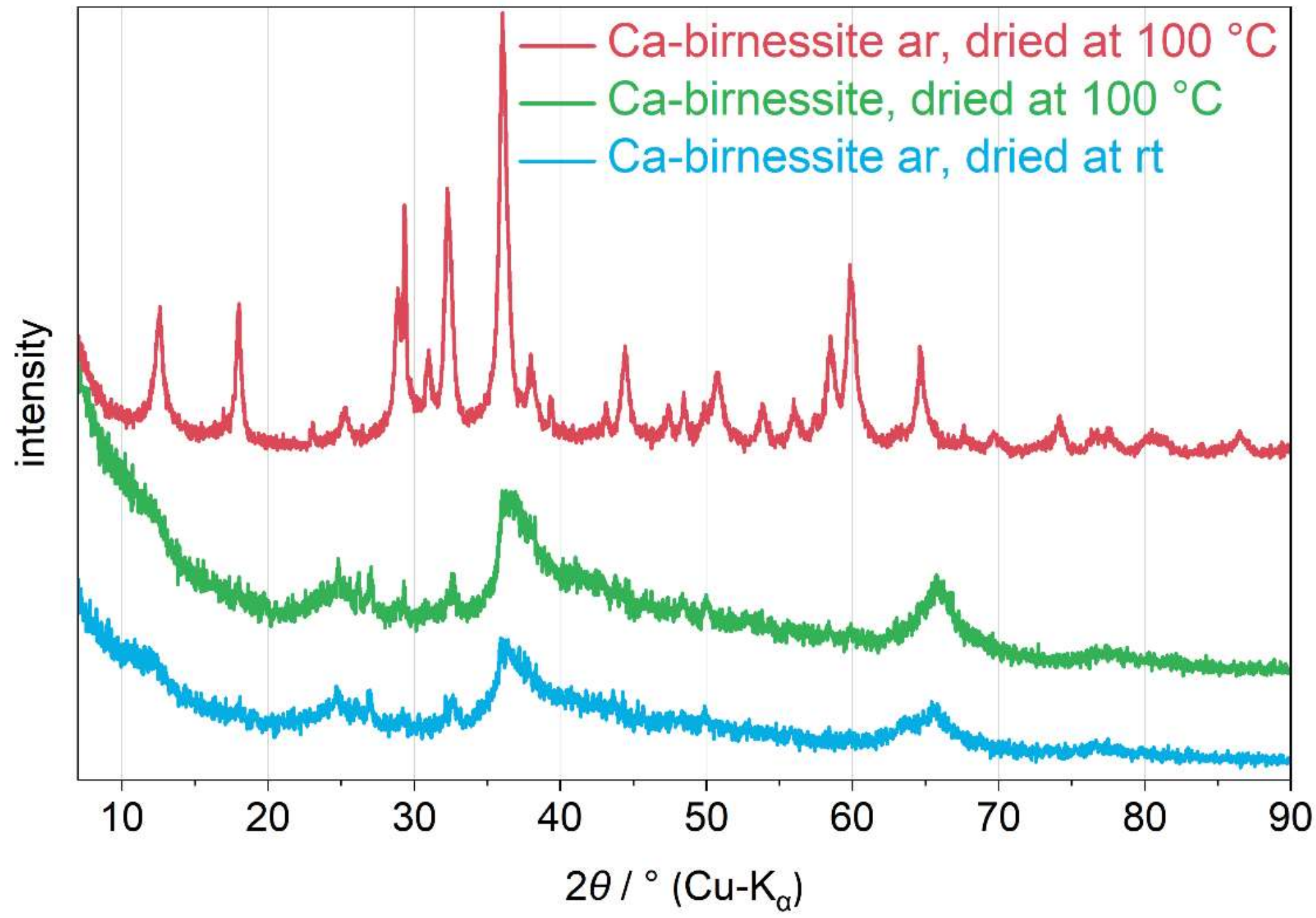

Figure S 19. pXRD patterns of the synthetic Ca-birnessite stirred in acetonitrile and then dried at 100 °C (bottom, in blue) compared to the diffractograms for the Ca-birnessite after its reaction with *trans*-pent-2-en-1-ol and dried either at room temperature (middle, in green) or at 100 °C (top, in red).

7.1. Additional Mn K-edge XAS comparison of pristine Ca-birnessite and mildly dried Ca-birnessite ar

To further evaluate the reaction-induced changes of Ca-birnessite, an additional Mn K-edge XAS comparison was performed between pristine Ca-birnessite and a mildly dried Ca-birnessite sample post-reaction. The XANES spectra show a moderate shift of the absorption edge for Ca-birnessite ar, consistent with partial Mn reduction after reaction. The FT-EXAFS spectra show that the first-shell Mn-O contribution is retained, while the birnessite-type edge-sharing Mn-Mn contribution is attenuated and broadened and additional intensity appears at slightly higher R′. The corresponding WT-EXAFS maps confirm that the main Mn-O/Mn-Mn scattering contributions remain in similar k-R′ regions, but with modified higher-shell intensity after reaction. These results support the interpretation in the main text that Ca-birnessite undergoes reaction-induced local disordering and partial spinel/hausmannite-like reorganization, while retaining residual birnessite-type motifs.

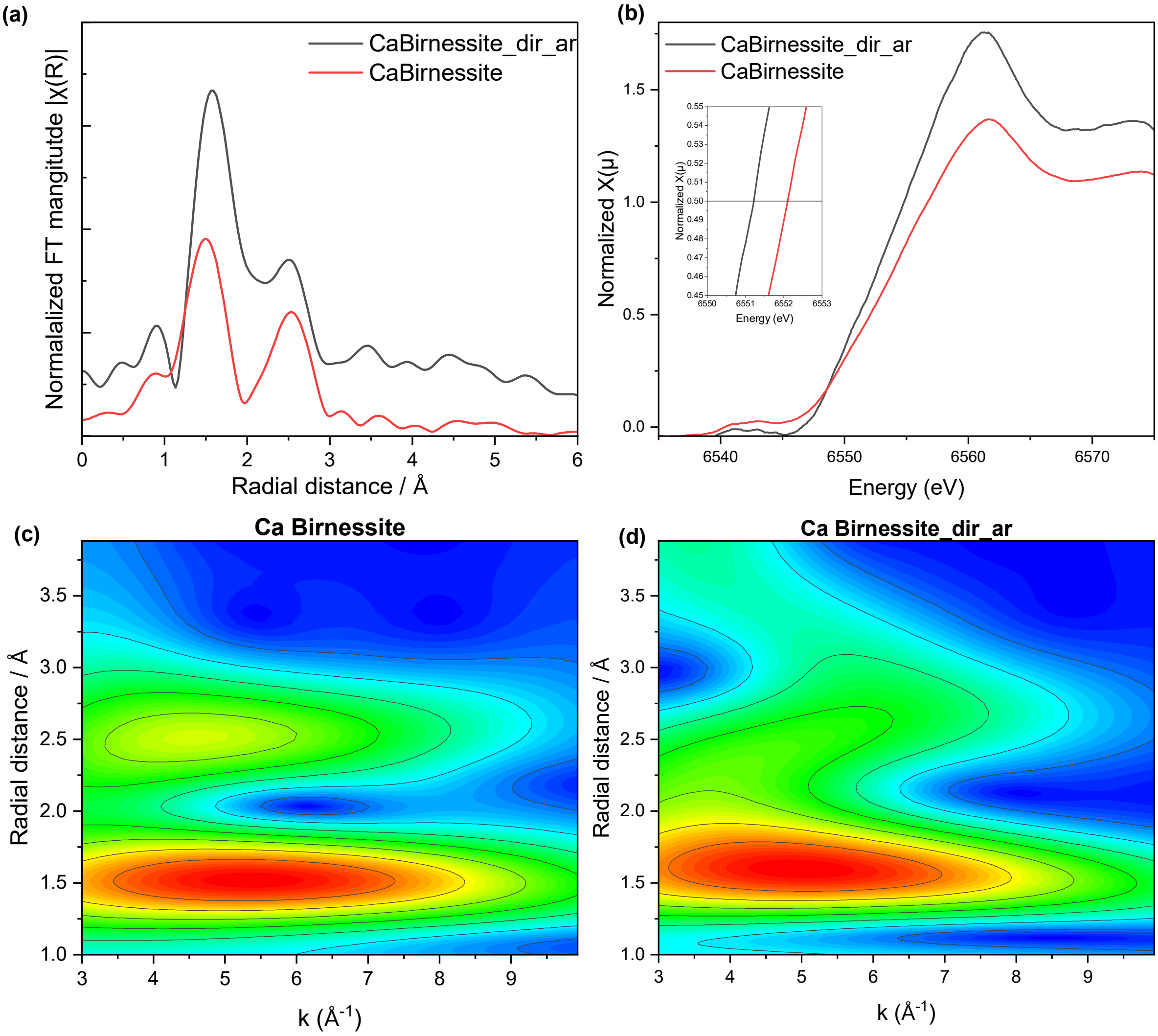

Figure S 20. Mn K-edge XAS comparison of pristine Ca-birnessite and mildly dried post-reaction Ca-birnessite ar. (a) Fourier-transformed EXAFS spectra plotted as |χ(R)| versus the phase-uncorrected radial distance R′. (b) Normalized XANES spectra with an inset highlighting the absorption-edge region. (c,d) Corresponding WT-EXAFS maps of pristine Ca-birnessite and Ca-birnessite ar, respectively; the radial-distance axis is also phase-uncorrected. The Ca-birnessite ar spectrum shows a moderate shift in the XANES edge region, consistent with partial Mn reduction after reaction. The FT-EXAFS and WT-EXAFS data show retention of the first-shell Mn-O contribution, attenuation and broadening of the birnessite-type Mn-Mn feature, and additional higher-R′ intensity after reaction, consistent with reaction-induced local disordering and partial spinel/hausmannite-like reorganization.

8. NMR evaluation of the oxidation of cinnamyl alcohol

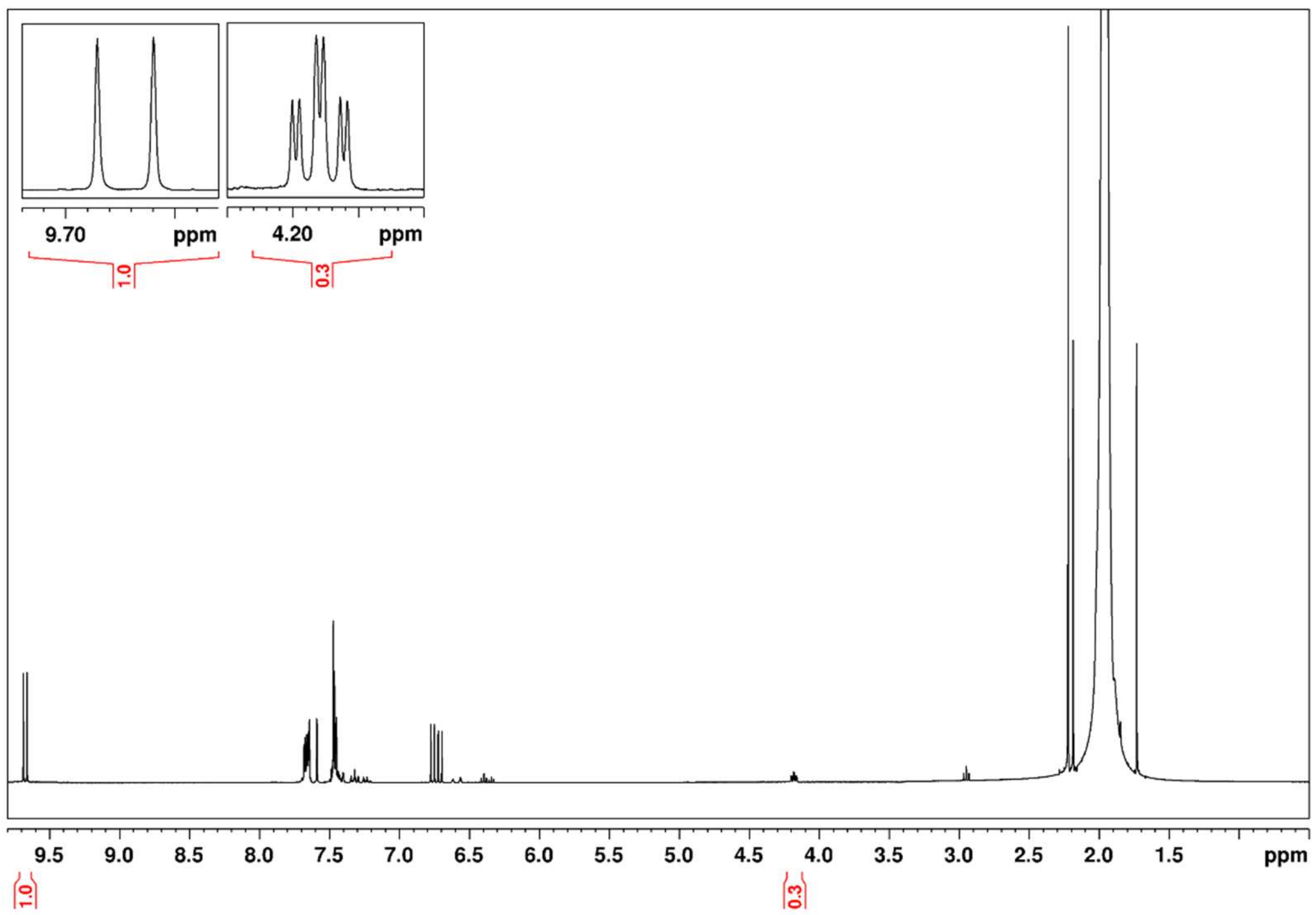

Figure S 21. ^{1}H NMR spectrum of a reaction mixture containing cinnamyl alcohol (*trans*-3-phenylprop-2-en-1-ol) and cinnamaldehyde (*trans*-3-phenylprop-2-en-1-al). Reaction conditions: 60 minutes reaction time in acetonitrile with Ca-birnessite as oxidation agent, Mn : substrate ratio 10 : 1.
